# Enhancing the Multifunctional Qualities of Palm Oil With Plant‐Derived Essential Oils: An Integrated HPLC and RSM Approach

**DOI:** 10.1002/fsn3.71741

**Published:** 2026-04-15

**Authors:** Manal A. Almalki, H. Uguru, O. Nyorere, O. Eboibi, O. Akpomedaye, Nashi K. Alqahtani, Sarah Alharthi, Rokayya Sami, Norah E. Aljohani, Afnan M. Alnajeebi, Mahmoud Helal

**Affiliations:** ^1^ Department of Chemistry, College of Science Taibah University Al‐Madinah Saudi Arabia; ^2^ Department of Agricultural Engineering Southern Delta University Ozoro Nigeria; ^3^ Department of Mechanical Engineering Southern Delta University Ozoro Nigeria; ^4^ Department of Electrical Engineering Southern Delta University Ozoro Nigeria; ^5^ Department of Food and Nutrition Sciences, College of Agricultural and Food Sciences King Faisal University Al‐Ahsa Saudi Arabia; ^6^ Department of Chemistry, College of Science Taif University Taif Saudi Arabia; ^7^ Research Center of Basic Sciences, Engineering and High Altitude Taif University Taif Saudi Arabia; ^8^ Department of Food Science and Nutrition, College of Sciences Taif University Taif Saudi Arabia; ^9^ Department of Clinical Nutrition Taibah University Medina Saudi Arabia; ^10^ Department of Biological Sciences, College of Science University of Jeddah Jeddah Saudi Arabia; ^11^ Department of Mechanical Engineering, Faculty of Engineering Taif University Taif Saudi Arabia

**Keywords:** essential oils, industrial oil applications, oil preservation, oxidation inhibition, physical properties

## Abstract

The major goal of this research was to develop a suitable model‐based optimization strategy that will preserve the bioengineering properties of palm oil by optimizing natural essential oils—precisely, turmeric oil (TUO), ginger oil (GO), and banana peel oil (BPO). The palm oil was blended by using a well‐structured experimental design based on optimal mixture and response surface methodology. All the oil samples were heated to 200°C for 150 min, and the antioxidant effect of the treatments on the thermal degradation of the samples was determined in accordance with standard procedures. The results revealed that the kinematic viscosity, flash point, carotenoids, and tocopherols of the fresh palm oil were 32.05 mm^2^/s, 319.74°C, 751.67 mg/kg, and 871.00 mg/kg, respectively. However, the extended exposure to high temperature caused the iodine value, kinematic viscosity, carotenoids, tocopherols, oxidative stability index, and breakdown voltage of untreated palm oil to decrease by 52.37%, 63.34%, 41.11%, 94.26%, 76.98%, and 65.23%, respectively. Also, based on the Combined I‐optimal mixture and response surface experimental design results, the treatments substantially inhibited the degradation of the PO, thereby preserving its quality. The anti‐thermal degradation ability of the EOs was further confirmed by the treated oil's BDV values, spectra, and DPPH values. After heating, the unfortified PO sample exhibited the maximum O–H and peak values of 3400 cm^−1^ and 1710–1720 cm^−1^, respectively; while the blended PO showed the minimum peak value of 2920 cm^−1^. Remarkably, the model developed in this study was able to predict the effectiveness of these treatments in improving the PO quality, as well as stabilizing these attributes in the presence of extended high temperatures. The model validation revealed that the PO samples blended with high concentrations of TUO yielded the optimal results, with consistent high retention levels of breakdown voltage, carotenoids, oxidative stability index, and tocopherols, as well as lower values of the free fatty acids and peroxides. Specifically, the hybridization results showed that the ranking of thermal stability effectiveness of the essential oils trailed this pattern: TUO> GO > BPO. Finally, the outcomes of this study will provide a scientific foundation for utilizing plant‐based essential oil to achieve more thermally stable vegetable oils, with enhanced dietary, engineering, and pharmaceutical applications.

## Graphical abstract

The results established that the treatments obviously retard the thermal degradation and hydrolytic decomposition of the palm oil, which can be attributed to the antioxidant effects (high phytochemical content) of the essential oils used as the treatment material. Markedly, the bio‐fortified palm oil samples recorded greater nutritional, physiochemical, and dielectric properties. Although the electrical transformer performance was not among the major objectives of this research, the breakdown voltage response further validates the treatments' resistance to thermal degradation.

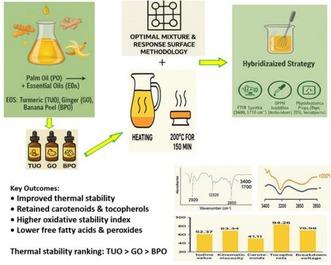

## 1 Introduction

Effective food preservation and storage are very essential in combating food insecurity, mortality and poverty, as food spoilage is one of the major causes of malnutrition and foodborne diseases. Apart from the food sector, adequate crop processing enhances its pharmaceutical and engineering properties, as these processed food items are either used in drug manufacturing or in the production of green energy (Amanullah et al. 2024; Chaachouay and Zidane 2024). Some processed forms of plants, including juice, oil, and extracts, have wide industrial applications. Plant juice is mainly used as a carrier agent in pharmaceuticals and also in bioethanol production, which is used in the food and electric power production industries. Additionally, plant extract is a suitable material for food preservation, nutraceuticals applications, and blending biofuels to increase their performance and stability (Karabulut and Goksen 2025). Vegetable oils (VOs) play major roles in the medical, nutritional, and engineering sectors, basically due to their high levels of physiochemical properties, bioactive compounds, and antioxidant activity (Uguru et al. 2023; Golodnizky et al. 2025).

Palm oil (PO), extracted from palm tree ( *Elaeis guineensis* ) fruits, is one of the most widely utilized oils both in the domestic and industrial sectors. It is edible due to its non‐toxicity and high nutritional content, and it is also used in the production of cosmetics, pharmaceutical products, soaps, coolant, and biofuels. Palm oil is subjected to chemical and oxidative degradation during storage operations, leading to the production of peroxides and free fatty acids, thereby reducing the oil's quality and integrity (Alhaji et al. 2024; Imam et al. 2025). During food processing and other industrial applications, PO is exposed to high temperatures that may cause thermal degradation, resulting in decline in the palm oil quality and applications, particularly nutritional values and dielectric properties (Šegatin et al. 2020; Tsai et al. 2023). Also, highly thermal oxidized (thermal hydrolysis) oil tends to accumulation of some toxic compounds, which have been attributed to cancer, cardiovascular diseases and kidney failure (Dodoo et al. 2022).

Blending of palm oil with suitable additives (usually essential oils) enhances its nutritional quality, stability, and industrial applications. These additives have the potential to modify and stabilize the oil's triacylglycerol profiles and other engineering properties. This results in the formation of improved PO with better nutritional values, fuel properties, textural qualities, and thermal stability (Ramroudi et al. 2022; Mahmud et al. 2023). Natural additives are usually preferred over synthetic additives because of the toxicity associated with consuming inorganic compounds in the food industry. Plant‐based additives are widely used in food preservation, primarily due to their rich antioxidants and antimicrobial properties, as well as non‐toxicity and environmental friendliness (Ali et al. 2025). Antioxidants can significantly inhibit the oxidative reaction of vegetable oil by interfering with the oxidation chain reaction occurring within the oil. This helps to avert the production of toxic oxidation products, hence preserving the oil's self‐life.

Numerous scholars have conducted research on the preservation and stability of vegetable oils' quality, mainly palm oil, particularly during storage and when exposed to prolonged high temperatures (Shahid et al. 2018; Ramroudi et al. 2022; Adiiba et al. 2023; Kedir et al. 2023). These investigations were mainly focused on a single or a combination of two natural additives. Also, several studies have investigated the use of more cost‐effective synthetic antioxidants, such as Butylated hydroxyanisole (BHA), tertiary butylhydroquinone (TBHQ), and butylated hydroxytoluene (BHT), to increase the oxidative stability and nutrient preservation in vegetable oils (Mika et al. 2023; Jyoti et al. 2025; Yan et al. 2025). These synthetic compounds have severe health implications, when accumulated in the body in large qualities (Eze et al. 2025). Therefore, due to the rapid pursuit of health‐friendly treatments, this research was conducted to evaluate the effectiveness of natural antioxidants (plant‐based additives) particularly in the food industry, rather than focusing on the direct cost implications of synthetic antioxidants. It has now become paramount to design a robust experiment that, will be able assess the collaborative impact of EOs concentration, and heating time on several PO quality indices. Precisely, these natural additives should potentially enhance the PO's shelf life, quality, nutrient preservation, and thermal stability when subjected to extended high temperatures, as well as, enhancing the usability of PO, while preserving its dielectric properties, as eco‐friendly lubricants and bio‐coolants in electrical transformers. The vital aim of this research is to evaluate how plant‐based essential oils (turmeric oil (TUO), ginger oil (GO), and banana peels oil (BPO)), can enhance the thermal stability and oxidative resistance of palm oil. Basically, this research assesses the efficacy of selected EOs in improving the thermal oxidative stability, physicochemical stability, and engineering properties of PO under extended high temperatures, which are relevant to applications in the food industry. Specially, the information obtained from this research will establish the bioeconomy of some agricultural waste materials, as these by‐products can be refined into functional additives.

## 2 Materials and Methods

### 2.1 Chemicals and Scientific Materials

The chemicals used for laboratory analyses were of analytical grade, mainly manufactured by Thermo Fisher Scientific Inc., based in America. The HPLC system with a column (10 μm, 3.9 mm × 300 mm) was produced by Thermo Fisher Scientific Inc., America. The electric blender (Model: KNW‐608) was manufactured by Kenwood Limited, England. The FTIR (Fourier Transform Infrared) spectrometer (Model: 4500 Series) was manufactured by Agilent Technologies, America. The digital water bath (Model: HH6) was manufactured by Whyzee Nigeria Limited. The digital capillary viscometer (Model: GD‐265C) was manufactured by Focus Technology Co. Ltd., China. The FTIR spectrometer (Model: Great 20) was manufactured by Changsha Lonroy Technology Co. Ltd., China.

#### 2.1.1 Basis for the Methodological Selection and Experimental Design

The practical framework for this research was intentionally designed to support the main goal of this study, which is to evaluate the effectiveness of turmeric oil, ginger oil, and banana peel oil in preventing the thermal degradation of palm oil when exposed to extended high temperatures. Though palm oil is already a fairly heat‐stable compound, the aim of blending it with more volatile EOs is not primarily to enhance the palm oil inherent thermal properties, but to inhibit (or suppress) thermal oxidative deterioration during prolonged exposure to high temperatures, especially in industrial applications. Furthermore, the rationale of selecting the turmeric rhizome oil (TUO), ginger rhizome oil (GO), and banana peel oil (BPO), in this study based on their diverse phytochemicals' contents. Turmeric rhizome oil is rich in curcumin; ginger rhizome oil contains gingerol and zingiberol; and banana peel oil contains polyphenols (Zhou et al. 2022; Mohd et al. 2022). The interaction of these essential phytochemical compounds will create a synergy that enhances the antioxidant mechanisms of the treatments. This will increase the thermal stability of the treated palm oil during exposure to prolonged high temperature.

Additionally, the lower TUO, GO, and BPO concentration ranges of 0 to 2.5% v/v were adopted in this experimental framework so that these essential oils will not have adverse effects on the sensory properties and anti‐nutrient levels of the fortified palm oil. Higher concentrations of essential oils in treatments tend to have some health implications and also cause sensory modification (Pezantes‐Orellana et al. 2024).

The RSM combined with the CCD was used to model and enhance the hybridized effects of treatment concentration and temperature exposure, with the aid of the Design‐Expert software. Collectively, these procedural choices were made to ensure consistency and repeatability, primarily between the research plan and the central research question. Additionally, apart from the oil deterioration during heat stress conditions, high temperatures cause serious alterations to the physiochemical properties of palm oil, which are essential for industrial applications. Hence, breakdown voltage (BDV), a dielectric parameter, was incorporated into the experimental design as one of the response variables. Though palm oil BDV is a crucial factor in electric transformer oil standards, its incorporation as one of the evaluated parameters serves only as a proportional indicator of potential changes in oil structure during prolonged heating.

### 2.2 Samples Preparation

#### 2.2.1 Source of Fresh Materials

The ripe palm fruits bunch was harvested from the oil palm plantation of the Southern Delta University, Ozoro, Nigeria. Each bunch was split, and the fruits were collected. The turmeric and ginger rhizomes were harvested from the experimental farm of Southern Delta University, Ozoro, Nigeria, planted purely under an organic farming method. Then, banana peels were collected from local food vendors in Delta State, Nigeria.

#### 2.2.2 Essential Oil (EO) Production

The turmeric and ginger rhizomes, as well as the banana peel, were chopped and sun‐dried (26°C–37°C) before being ground using a laboratory blender. The oil content of the different samples was extracted using the solvent extraction technique, with *n*‐hexane as the solvent. 100 g of the sample was poured into a thimble, inserted into a Soxhlet extraction apparatus, and 1 L of *n*‐hexane was added to the system. The mixture was then heated at 60°C for 420 min. Thereafter, the crude extracted oil was dried in a water bath at 45°C to evaporate the *n‐*hexane residue (Elouafy et al. 2022). This low temperature was chosen to prevent the oxidation and degradation of the essential oil produced.

#### 2.2.3 Palm Oil (PO) Production

The palm oil was extracted through a cold‐press method. The ripe palm fruits were manually selected and washed with borehole water to discard the premature and pest‐infested ones, after which the pulp was extracted from the fruits using a local de‐pulper. This pulp was crushed to release the oil from the fibrous tissue, and the product was transferred into an oil extractor at room temperature (26°C–32°C). The impurities in the crude PO were allowed to settle at the bottom of the container where they were drained away, and the remaining oil was dried at 40°C in a water bath for 60 min.

The oil yield was calculated using Equation 1, and the oil produced was poured into dark glass bottles and stored in a dark, air‐conditioned environment at 15°C ± 2°C prior to their utilization.

(1) $$\text{Oil Yield}\;\left(\%\right)=\frac{W_{1}}{W_{2}}\times 100$$

where: W_1_, mass of oil produced; W_2_, mass of the material used.

### 2.3 Experimental Design and Laboratory Analyses

The Response Surface Methodology (RSM), which uses the basics of the Central Composite Design (CCD), was utilized to develop the model and also to optimize the influence of the treatment concentration and heating duration on the PO dependent variables. Design‐Expert was used to generate the models, perform statistical analysis, as well as the optimization strategies. Particularly, this design has 28 experimental runs, which were replicated three times. The two independent variables in this study were EO concentration (v/v) and heating duration (minutes), each with six levels considered. Specifically, the heating duration ranged from 0 to 150 min (30 min per interval), and the EO concentration ranged from 0% to 2.5% (0.5% per interval). Then, the dependent variables were antioxidant activity, oxidative stability index (OSI), peroxide value (PV), free fatty acid (FFA), iodine value (IV), carotenoids, tocopherols, specific viscosity, and flash point. All the models' reliability were scrutinized with different parameters, which include *R* ^2^, adjusted *R* ^2^, forecasted *R* ^2^, Adeq precision, and lack‐of‐fit tests, as well as additional experiments. Then, the findings were compared with the predicted response values to ascertain whether the results did not exceed the predicted ranges, thereby confirming the models' reliability.

The heating temperature was set constant at 200°C throughout the experimental duration, to prevent confounding resulting from temperature instabilities. This temperature was taken based on information from previous research (Nduka et al. 2021; Poudel and Kusma 2025), and it exemplifies consistent high‐stress, as most palm oil thermal exposure—deep‐frying and industrial heating operations have temperatures that fall within the range of 200°C (Juvvi et al. 2024). Additionally, the EOs concentration (0%–2.5% (v/v)) was chosen based on information from well‐documented literature (Zhao et al. 2021; Yeasmin et al. 2024; Uguru et al. 2023). Basically, these lower concentration levels were used to avoid interference of the plant‐derived essential oils with the sensory properties of palm oil, and to prevent other pro‐oxidant effects. Also, the three EOs (TUO, GO, and BPO) were chosen based on their proven high antioxidant content: curcuminoids, gingerols, and catechins (Orellana‐Paucar 2024; Ajanaku et al. 2022; Maghraby et al. 2023). These antioxidants will play dual functions, enhancing the PO quality and inhibiting the thermal degradation of the oil.

#### 2.3.1 Laboratory Analyses

Laboratory analyses were carried out in two sets of palm oil. One set was analyzed to determine the impact of high temperature on the quality of unblended PO; while the other set was analyzed to evaluate the impact of blending agents (EOs) on the PO quality during heating. These findings will give clear information on the effects of high temperature on PO quality, as well as the roles played by blending agents in stabilizing PO quality during prolonged exposure to high temperatures. Basically, all the calibration parameters used for the phytochemicals determination are presented in Table S1.

### 2.4 Oxidation By‐Product Determination

#### 2.4.1 Free Fatty Acid (FFA)

The FFA level in the oil was determined using the AOAC (2023) official approach, via titration technique. Sodium hydroxide was used as the base, and phenolphthalein as the indicator. The FFA content of each sample was then calculated using Equation 2.

(2) $$\mathrm{FFA}=\frac{V\times N\times 28.2}{W}$$

where: V, volume of base used; N, normality of the base; W, weight of PO used.

#### 2.4.2 Peroxide Value (PV)

The oil PV was determined through the titration method, by following the AOAC (2023) official procedure. 10 g of the PO was poured into a conical flask, and then 60 mL of a mixture of CH_3_COOH and CHCl_3_in a ratio of 6:4 was added. Then, 1 mL of potassium iodide solution was introduced into the mixture and allowed to stand for 5 min in a dark environment. Thereafter, 60 mL of distilled water was added to the resulting product, which was titrated with 0.01 N Na_2_S_2_O_3_solution till the yellow coloration disappeared. Then added another 1 mL of starch solution (1%), and titrated again until the blue coloration faded. The oil PV was calculated using Equation 3 (Nduka et al. 2021).

(3) $$\mathrm{PV}=\frac{\left(A-B\right)\times N\times 1000}{W}$$

where *A*, Na_2_S_2_O_3_ volume (mL); *B*, Na_2_S_2_O_3_ volume used during the blank titration; N, Na_2_S_2_O_3_ normality; *W*, oil weight (g).

### 2.5 Physical–Chemical Index Determination

#### 2.5.1 Iodine Value (IV)

The oil's IV was determined by using the Wijs method, by following the AOAC (2023) official approach. 0.4 g of the PO was dissolved in a mixture of 10 mL of CHCl_3_ and 25 mL of Wijs solution. The product was left for 30 min, before it was added to 20 mL of potassium iodide solution (10%) and 100 mL of distilled water. This was titrated with 0.1 N Na_2_S_2_O_3_ until the yellow color disappeared. Then, another 2 mL of 1% starch solution was added to the already titrated product, and the solution was titrated again until the blue coloration faded. The iodine content of the oil was calculated using Equation 4, and expressed as g I_2_/100 g oil (Nduka et al. 2021).

(4) $$\mathrm{IV}=\frac{\left(A-B\right)\times N\times 12.69}{W}$$

#### 2.5.2 Kinematic Viscosity

A digital capillary viscometer was used to determine the kinematic viscosity of the PO samples, in accordance with ASTM D445 procedures at 40°C ± 0.5°C. The oil kinematic viscosity (ν) value was calculated via Equation 5 and expressed as mm^2^/s.

(5) v=K×t

where: K, viscometer constant (0.35 mm^2^/s^2^); and t, time (s).

#### 2.5.3 Flash Point Determination

The oil sample's flash point was measured using the Cleveland open cup technique in accordance with ASTM D92 procedures. 100 mL of the sample was sieved, poured into the test cup, and heated gently. A control flame test was conducted at every 2°C increment, and the lowermost temperature at which a flash occurred was taken as the sample's flash point.

#### 2.5.4 Oxidative Stability Index (OSI) Determination

The oxidative stability index of the samples was determined by following the AOCS (2017) official method. 5 g of the sample was measured into the Rancimat reaction vessel, which was connected to a heating block. The following parameters were used to determine the oil OSI: operating temperature—117°C, airflow rate—20 L/h, and water volume—70 mL. At the end of each experiment, the electronic section of the system computed the OSI values and displayed the results, which are expressed in hours.

#### 2.5.5 Break Down Voltage (BDV) Determination

The samples' BDV was determined using the oil dielectric tester in accordance with ASTM D1816 procedures. The sample was filtered and poured into the test cell of the apparatus, and then the electrode gap was adjusted to a 1 mm clearance. A gradual AC voltage was applied to the unit until the sample experienced a breakdown. The BDV was computed using Equation 6 and expressed as kV.

(6) $$\mathrm{BDV}=\frac{\sum \text{breakdown voltages}}{\text{number of tests}}$$

### 2.6 Bioactive Compounds Determination

#### 2.6.1 Tocopherol Determination

The tocopherols level in the samples was determined through the AOAC (2023) standard, and by HPLC profiling, using a reverse‐phase C18 column and UV detection. 0.5 mL of the sample was diluted with 10 mL of *n*‐hexane, then mixed thoroughly and sieved through a 0.45 μm filter. 20 μL of the solution was injected into the HPLC vial, and the tocopherol content in the sample was measured using these HPLC parameters: flow rate—1.0 mL/min, wavelength—292 nm, column temperature—25°C, retention time—15 min, and run time—29 min.

#### 2.6.2 Total Carotenoids Determination

The UV–Vis spectrophotometer was used to measure the total carotenoids in the samples, by adopting the AOAC (2023) guidelines. A 0.5 mL of the sample was added to 50 mL of *n‐*hexane solvent. The cuvette was filled with the prepared solution, inserted into the system, and the absorbance was measured at 446 nm. The sample's total Carotenoids level was calculated using Equation 7, and expressed as mg/kg.

(7) $$\text{Total Carotenoids}=\frac{A\times 10^{4}}{2592}$$

where *A* = absorbance rate at 446 nm.

#### 2.6.3 Alkaloid Determination

The alkaloid content was determined using the spectrophotometric approach. 2 mL of EO was added to 10 mL bromocresol green solution (prepared by mixing 69.8 mg of bromocresol green with 10 mL of phosphate buffer) and 10 mL chloroform, and the mixture was centrifuged at 2500 rpm for 5 min. Then, the lower stratum was collected, and its absorbance was measured with a spectrophotometer at 470 nm. Specifically, chloroform was used as the blank material.

#### 2.6.4 Saponins Determination

The HPLC method was used to measure the saponins content. 20 μL of the filtered EO was injected into the HPLC system with a UV detector, which uses the C18 column. The system operates at a 1 mL/min flow rate, column temperature of 27°C, methanol mobile phase, wavelength of 210 nm, and run time of 55 min (Fordos et al. 2025).

#### 2.6.5 Total Phenolic Content (TPC) Determination

The essential oils TPC levels were measured using the UV–Visible spectrophotometry approach. Gallic acid was used as the benchmark, and the spectrophotometer was set at 760 nm. 1 mL of the EO was mixed with 5 mL of Folin–Ciocalteu reagent (prepared by diluting one part of Folin–Ciocalteu with 10 parts of deionized water). The resulting mixture was incubated at 27°C for 25 min before its absorbance at 760 nm, and TPC computed using the expression in Equation 8 (Shahid et al. 2018).

(8) $$\mathrm{TPC}=\frac{\text{mgGAE}}{g}$$

where GAE, gallic acid equivalent.

#### 2.6.6 Total Flavonoid Content (TFC)

The samples TFC were determined by using UV–Vis Spectrophotometer approach. 0.5 mL of the sample was mixed with 2.8 mL of distilled water, 0.5 mL of ethanol, 0.1 mL of 10% AlCl_3_(_aq_), and 0.1 mL of 1 M KAc. The product was kept at 25°C for 25 min, before its absorbance was taken at 415 nm. Yhe TFC level in each sample was calculated with Equation 9, with quercetin equivalent (QE)/gram unit.

(9) $$\mathrm{TFC}=\frac{\text{mgQE}}{g}$$

where: QE, quercetin equivalent.

### 2.7 In Vitro Antioxidant Activity Through DPPH Inhibition

The DPPH solution was prepared by dissolving 10 mg of DPPH in 100 mL of methanol, and 1 mL of the solution was mixed with 1 mL of the PO sample. Thereafter, the resulting product was incubated at 24°C for 30 min before its absorbance was measured at 517 nm. Similarly, a control was prepared by mixing DPPH solution with methanol (no antioxidant) at a 1:1 ratio. Consequently, the DPPH radical scavenging activity of the sample was calculated through Equation 10 (Brand‐Williams et al. 1995).

(10) $$\text{DPPH Scavenging Activity}\;\left(\%\right)=\frac{A_{C}-A_{S}}{A_{C}}\times 100$$

where: A_C_ and A_S_ are the DPPH solution absorbance of the control and tested sample, respectively.

### 2.8 FTIR Analysis

The sample's FTIR analysis was conducted in accordance with ASTM E1252 standard procedure. It uses an FTIR spectrometer with wave number accuracy ≤ 0.01 cm^−1^, at a laboratory temperature of 27°C ± 2°C. 1 mL of the sieved specimen was placed unto a diamond ATR crystal to emit different spectra, which were scanned over the range of 4000–400 cm^−1^ at 4 cm^−1^ resolution (Dodoo et al. 2022).

### 2.9 Statistical Analysis

The results were scrutinized using the ANOVA tool to evaluate the model significance (*p* < 0.05). Specifically, the fit of the model was assessed using the *R* ^2^, adjusted *R* ^2^, and lack‐of‐fit tests. Also, the Design‐Expert was utilized for RSM‐based analysis. The one‐way ANOVA was used to establish the effect of the heating period on the palm oil quality, while DMRT was used to separate the means. The tests were conducted in triplicate, while the optimization was carried out by adopting the desirability function method.

## 3 Results and Discussion

### 3.1 Essential Oils Bioactive Compounds

The results of the yields of the essential oils and their respective phytochemicals, as measured, are presented in Table 1. It was observed that TUO had the highest oil yield, while BPO had the lowest, affirming previous assertion that plant genotype substantially affects EO's yield (Hazrati et al. 2022). The findings showed that BPO had the highest levels of alkaloids and saponins; TUO exhibited the highest levels of TPC, TFC, and curcumin; while GO had the highest contents of gingerol and zingibero. These essential phytochemicals found in these three EOs have strong antioxidant effects, enhancing the thermal stability of the palm oil, which may result in improved application effectiveness. The bioactive compounds found in plant‐based antioxidants retard the oxidation of oil molecules during high temperatures, thereby preserving the oil's nutritional and fuel properties (Fordos et al. 2025; Montoro‐Alonso et al. 2025; Singh et al. 2025).

**TABLE 1:** The essential oils properties.

Parameter	TUO	GO	BPO
Yield (%)	9.62^c^ ± 0.89	6.87^b^ ± 0.60	4.23^a^ ± 0.55
Alkaloids (mg/g)	15.55^b^ ± 2.10	6.51^a^ ± 2.94	42.39^c^ ± 4.28
Saponins (mg/g)	28.02^b^ ± 4.03	9.12^a^ ± 2.70	57.05^c^ ± 7.85
TPC (mg GAE/g)	50.56^b^ ± 2.31	25.60^a^ ± 2.32	26.96^a^ ± 1.56
TFC (mg QE/g)	35.41^b^ ± 3.87	14.22^a^ ± 4.53	31.87^b^ ± 2.52
Gingerol (μg/mL)	NR	750.33 ± 89.19	NR
Curcumin (μg/mL)	36258.67 ± 967.12	NR	NR
Zingiberol (μg/mL)	NR	557.00 ± 22.61	NR

*Note:* For the same parameter columns having similar letter are not significantly different (*p* ≤ 0.05).

Abbreviations: BPO, banana peel oil; GO, ginger oil; NR, not read; TFC, total flavonoid content; TPC, total phenolic content; TUO, turmeric oil.

Furthermore, phenolic compounds and flavonoids are polyphenols that have perfect antioxidant effects. Therefore, a high concentration of these compounds will increase the ability of the treatments to scavenge radicals, leading to a reduction in peroxide value and polymer production during heating, as well as decreasing the oil's kinetic viscosity at high temperatures (Zahra et al. 2024). These active compounds of the essential oils play significant roles in their oxidative stability and enhance their applications in medical and engineering disciplines. The hybridized blending agents have the potential for multifunctionality due to the amalgamation of the macro and trace amounts of essential compounds they contain. Thereby, this enhances their relevance in various interdisciplinary applications and contributes to sustainable technologies.

### 3.2 Impact of Heating on the Unblended Palm Oil Properties

The results of the unblended palm oil (UPO) properties, before and after heating at 200°C for 150 min, are presented in Table 2. The heating caused a significant negative impact on the PO quality, as the essential compounds deteriorated significantly after the heating process. There was an increase in the PO's FFA and PV contents, whereas the IV, KV, OSI, BDV, flash point, carotenoids, and tocopherols decreased drastically after the heating duration. This observation is in conformity with the previous reports of Ramroudi et al. (2022) and Dodoo et al. (2022), where high temperatures have a negative impact of protracted heat on the physicochemical properties of vegetable oils. Though palm oil is stable, subjecting oils to prolonged elevated temperatures may result in the production of excessive FFA and hydroperoxides, primarily from triglyceride thermal oxidation (Jadhav et al. 2022; Kaur et al. 2020; Wang et al. 2023). This study's results corroborated the previously documented findings of Tsai et al. (2023) and Ebenezer and Aba (2024). Though Nduka et al. (2021) reported that high temperature (180°C) and shorter heating periods (≤ 7 h) exhibited no significant impact on the FFA value of untreated palm oil; this finding was contrary to this study's reports.

**TABLE 2:** The properties of the unblended palm oil.

Parameter	Fresh oil (Before heating)	Heated oil (After 150 min heating)
Yield (%)	27.33 ± 3.76	—
Free fatty acid (mg KOH/g)	2.33^a^ ± 0.21	5.22^b^ ± 0.15
Peroxide value (meq O_2_/kg)	3.10^a^ ± 0.24	6.10^b^ ± 0.07
Iodine value (g I_2_/100g)	65.72^a^ ± 1.46	31.30^b^ ± 1.83
Kinematic viscosity (mm^2^/s)	32.05^b^ ± 1.64	11.75^a^ ± 1.30
Flash point (°C)	319.74^b^ ± 3.14	210.27^a^ ± 6.63
Carotenoids (mg/kg)	751.67^b^ ± 11.37	442.67^a^ ± 13.05
Tocopherols (mg/kg)	871.00^b^ ± 1.14	486.67^a^ ± 13.43
OSI (hours)	45.75^b^ ± 1.99	10.53^a^ ± 1.01
BDV (kV)	35.00^b^ ± 1.22	12.17^b^ ± 1.62
DPPH (% inhibition)	52.50^b^ ± 2.92	17.79^b^ ± 1.75

*Note:* Columns having similar letter in the same parameter signifies no significantly difference between the means (*p* ≤ 0.05).

Abbreviations: BDV, break down voltage; OSI, Oxidative stability index.

Furthermore, the heating process depletes the oil's natural antioxidants, primarily the vitamins, leading to a reduction in its DPPH scavenging capacity. Tocopherols and carotenoids are essential compounds, which play some antioxidant mechanisms in vegetable oils. Hence, their depreciation exposes the palm oil to rapid oxidation (Athanasiadis, Chatzimitakos, et al. 2024; Athanasiadis, Kalompatsios, et al. 2024; Yildiz et al. 2025). High temperatures lead to the formation of larger amounts of lower weight volatile compounds, resulting in oil with a lower flash point. Flash point is a critical property of palm oil, which is linked to its industrial applications, mostly as a lubricant in engines or electrical power transformers (Khoirudin Kristiawan et al. 2024). Oil having an elevated flash point and BDV tends to be more thermally stable, possess effective cooling ability, reduces frictional force, and safer to handle, thereby enhancing the engine performance (Islam Sazzad et al. 2024; Romero et al. 2024).

### 3.3 Impact of the Treatments on the Palm Oil Qualities

The results of the effects of treatment concentration and heating time, on palm oil quality are presented in Table 3. The results have shown that, the incorporation of the additives was able to inhibit the oxidation and hydrolysis reactions of the PO, resulting in thermal stability of palm oil during heating. These findings can be associated with the divergent antioxidant mechanisms, thermal characteristics, and the concentrations of bioactive compounds in the essential oil (Table 1). This study's findings align with the report by Kedir et al. (2023), and Saragih et al. (2023), which stated that antioxidants have a very strong ability, to inhibit peroxide formation and trans fatty acid formation when palm oil is subjected to consistent heating. This study's results further corroborate Hao et al. (2019) observations, which stated that antioxidants positively influence the insulating behavior and electrical properties of oil under undergoing heat stress. The stability of carotenoids and tocopherols noted in the enriched PO samples aligns with the findings of Yildiz et al. (2025) and Nagy et al. (2024). According to Athanasiadis, Chatzimitakos, et al. (2024) and Athanasiadis, Kalompatsios, et al. (2024), treating PO with soybean oil and corn ( *Zea mays* ) oil enhances the oil's oxidative stability. The OSI and IV stability documented in the treated PO further confirmed its resistance to rancidity at high temperatures, which are influenced by the presence of phytochemicals (Kedir et al. 2023; Chabni et al. 2024). Carotenoids and tocopherols are essential antioxidants used to treat some cancers and neurodegenerative disorders, and they are also used in managing macular degeneration, DNA damage, and atherosclerosis (Adiiba et al. 2023; Tufail et al. 2024). Particularly, this study has tried to eliminate the deterioration of carotenoids and tocopherols during the high‐temperature frying process, as observed by previous researchers (Joshua 2023; Abrante‐Pascual et al. 2024; Egbung et al. 2024).

**TABLE 3:** Combined I‐optimal mixture and response surface experimental design for palm oil storage quality optimization.

Design point	TUO (%)	GO (%)	BPO (%)	Time (minutes)	PV (meq O_2_/kg)	FFA (mg KOH/g)	IV (g I_2_/100g)	Carotenoids (mg/kg)	Tocopherols (mg/kg)	KV (mm^2^/s)	ISO (h)	BDV (kV)	FP (°C)
1	1.97	2.03	0.00	90	4.01	3.26	58.92	650.27	694.15	20.56	35.83	23.44	273.18
2	0.00	2.01	1.99	0	3.53	2.44	65.40	750.44	823.82	35.34	43.21	33.63	311.34
3	2.00	0.00	2.00	150	4.22	4.12	47.06	525.22	548.93	15.37	20.74	15.36	235.84
4	0.00	2.03	1.97	90	4.25	3.32	57.11	661.24	683.41	21.15	32.15	22.18	279.96
5	0.00	0.00	4.00	150	4.11	4.25	40.92	518.04	552.58	16.48	17.34	13.77	245.84
6	0.00	2.01	1.99	150	4.03	4.13	42.18	522.12	542.93	15.83	18.77	14.36	240.16
7	0.00	2.03	1.97	90	4.25	3.32	57.11	651.99	683.41	21.15	32.15	21.99	279.96
8	1.95	0.07	1.97	60	3.85	2.77	64.23	698.27	755.06	25.38	40.87	28.35	285.93
9	1.97	2.03	0.00	90	4.01	3.26	58.93	650.27	694.15	21.00	35.83	23.44	274.00
10	0.61	0.72	2.67	30	3.78	2.68	64.76	722.24	800.12	31.28	41.87	33.24	290.77
11	2.00	2.00	0.00	0	3.48	2.45	65.39	751.09	823.89	34.61	45.95	36.11	303.28
12	0.00	4.00	0.00	90	3.86	3.18	62.19	675.39	692.00	21.95	36.61	22.97	277.59
13	0.00	0.00	4.00	60	3.91	2.96	63.58	719.66	769.38	27.03	42.03	26.06	290.03
14	4.00	0.00	0.00	0	3.59	2.42	65.44	750.45	822.11	32.13	50.63	37.94	308.25
15	1.95	0.07	1.97	60	3.85	2.77	64.23	700.00	758.93	25.11	40.97	28.44	285.93
16	0.64	2.70	0.65	120	4.31	3.79	51.38	526.01	624.94	17.38	26.85	19.21	250.62
17	0.00	4.00	0.00	150	3.95	4.19	43.57	548.75	568.55	14.03	19.85	14.92	238.47
18	0.69	0.66	2.65	120	4.39	3.91	49.61	548.82	612.53	18.77	24.41	16.85	258.29
19	0.00	4.00	0.00	0	3.63	2.41	65.42	751.44	824.92	33.75	48.55	36.82	300.17
20	4.00	0.00	0.00	60	3.79	2.58	64.73	725.35	788.18	24.62	44.48	30.25	280.84
21	2.01	1.99	0.00	150	3.85	4.06	42.95	555.93	562.74	15.36	20.38	17.17	239.93
22	0.00	0.00	4.00	0	3.68	2.44	65.40	752.49	823.86	34.95	46.84	32.16	307.56
23	4.00	0.00	0.00	150	3.82	3.87	45.11	568.47	588.37	14.98	22.53	17.99	236.14
24	2.66	0.69	0.64	30	3.82	2.59	64.98	737.97	810.66	30.89	42.36	34.16	279.54
25	0.65	2.69	0.66	30	3.89	2.65	64.57	729.85	803.25	30.13	45.39	33.89	288.18
26	1.99	0.00	2.01	0	3.65	2.47	65.39	750.08	825.27	24.82	47.26	35.57	303.14
27	1.95	0.07	1.97	60	3.87	2.75	64.23	715.11	757.47	25.76	40.85	28.32	287.15
28	1.97	2.03	0.00	90	4.04	3.28	58.93	650.13	695	20.25	36.02	23.95	272.79

Abbreviations: FP, flash point; ISO, oxidative stability index; IV, Iodine value; KV, kinematic viscosity.

Curcumin, gingerols, shogaols, and catechins, present in the treatments can effectively retard lipid peroxidation, by free radical quenching and membrane stabilization actions, thereby displaying potent anti‐hydroperoxide activity and leading to lower PVs and FFA formation during heating (Ajanaku et al. 2022; Gulcin 2025). Terpenoids and phenolic aldehyde compounds, which are secondary metabolites of the treatments, play an essential role by reducing lipid peroxidation during heat stress situations, which is linked to their membrane fortification ability (Gutiérrez‐Del‐Río et al. 2021). Additionally, flavonoid and phenolic compounds interfere with the radical chain mechanisms that facilitate oil oxidation, thereby improving and preserving the oil's oxidative stability (Gharby et al. 2025; Yildiz et al. 2025). Curcumin and tannins have strong metal‐chelating capabilities (binding pro‐oxidant heavy metals); hence, stabilizing the oil's breakdown voltage, kinematic viscosity, and oxidative stability index during heating (Smirnova et al. 2023). Antioxidants tend to deactivate singlet oxygen and impede lipoxygenase activity, leading to anti‐oxidation effects (Gulcin 2025), and this can be attributed to the higher quality of the enriched PO samples. Remarkably, the abovementioned mechanistic actions of the bioactive compounds, present in the treatments, will contribute substantially to stabilizing the oil's structural reliability and physicochemical properties during heat stress.

Breakdown voltage is one of the essential dielectric‐based physicochemical properties of palm oil. Therefore, higher values in the enriched POs, suggest lower formation of dipolar molecules and FFA during the heating duration. The response of the BDV to the treatments in this study aligns with the PV, FFA, OSI, and FTIR findings; therefore it buttressed the anti‐thermal degradation effects of the EOs used as treatments. Though this study does not explicitly investigate the electrical properties of palm oil, based on ASTM transformer oil specifications; the BDV values obtained revealed that enriched PO has dielectric performance, which could be consistent with ASTM D1816 specifications for bio‐coolant. The ASTM D1816 stated that bio‐coolant should exhibit a BDV value greater than 30 KV, which it should perfectly retained under high temperature (Oparanti et al. 2024; Nugroho et al. 2024). Interestingly, the ranking of thermal stability effectiveness, and DPPH inhibition ability, of three plant's oils followed this pattern: TUO>GO > BPO. The variations observed in the results obtained in this study, compared to other authors, can be directly linked to the concentrations of different bioactive compounds in the treatments, treatment compatibility with PO, and methodological approaches (Singh et al. 2025).

#### 3.3.1 DPPH Radical Scavenging Activity

**FIGURE 1:**
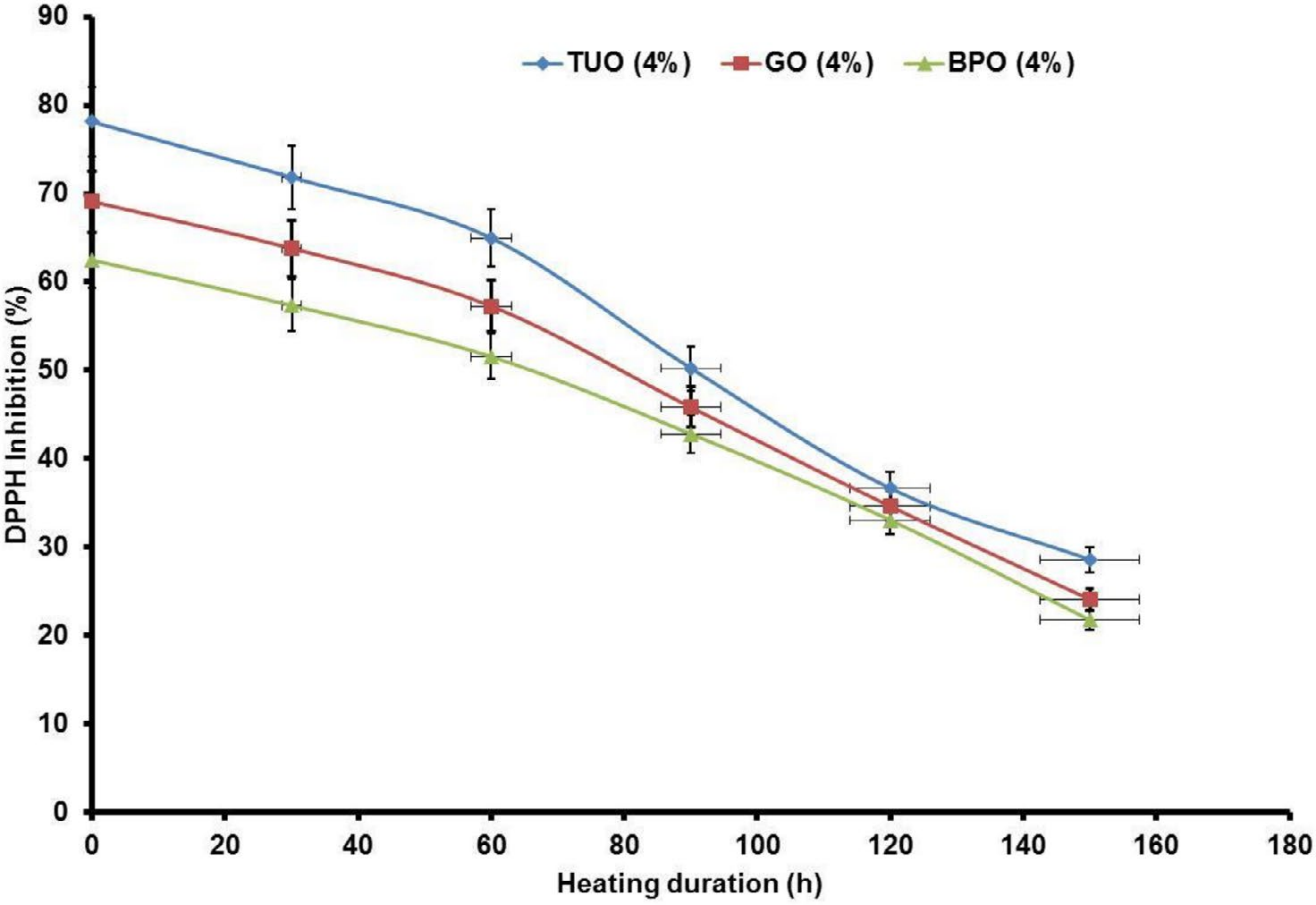
The impact of the blending agents on the PO DPPH inhibition.

Figure 1 shows the impact of the different essential oils on the enriched palm oil DPPH radical scavenging activity. It was noted that DPPH levels of the blended oil declined non‐linearly throughout the heating period. Specifically, the DPPH values recorded for the enriched PO after heating were higher than the value obtained for the unblended PO (17.79%), as shown in Table 2. DPPH inhibition ability of the essential oils could be linked to their excellent hydrogen‐donating potential, leading to their strong capacity to alleviate oxidative stress (Kedir et al. 2023; Gulcin 2025). The results portrayed that the TUO has the highest radical quenching activity, maintaining the same throughout the heating process. This can be attributed to the high concentrations of active antioxidants in the TUO (Table 1).

Contrary to earlier studies, whose results are mainly based on confirmatory analysis of the impact of essential oils on vegetable oils, this study integrates three aspects—EO hybridization, optimization, and model validation (Table 6)—to analytically explain the importance of green essential oils on palm oil. Generally, the results of this scientific investigation have established that the inclusion of bio‐based antioxidants into PO facilitates the retention of its bioactive compounds and natural antioxidants. Consequently, this enhances its shelf‐life, improves its physicochemical properties, and thermal stability during exposure to heat applications. This promotes the formation of more stable palm oil, with appreciable health‐promoting attributes, which have high prospects in the electrical, nutritional and biomedical industries.

### 3.4 FTIR Analysis

**FIGURE 2:**
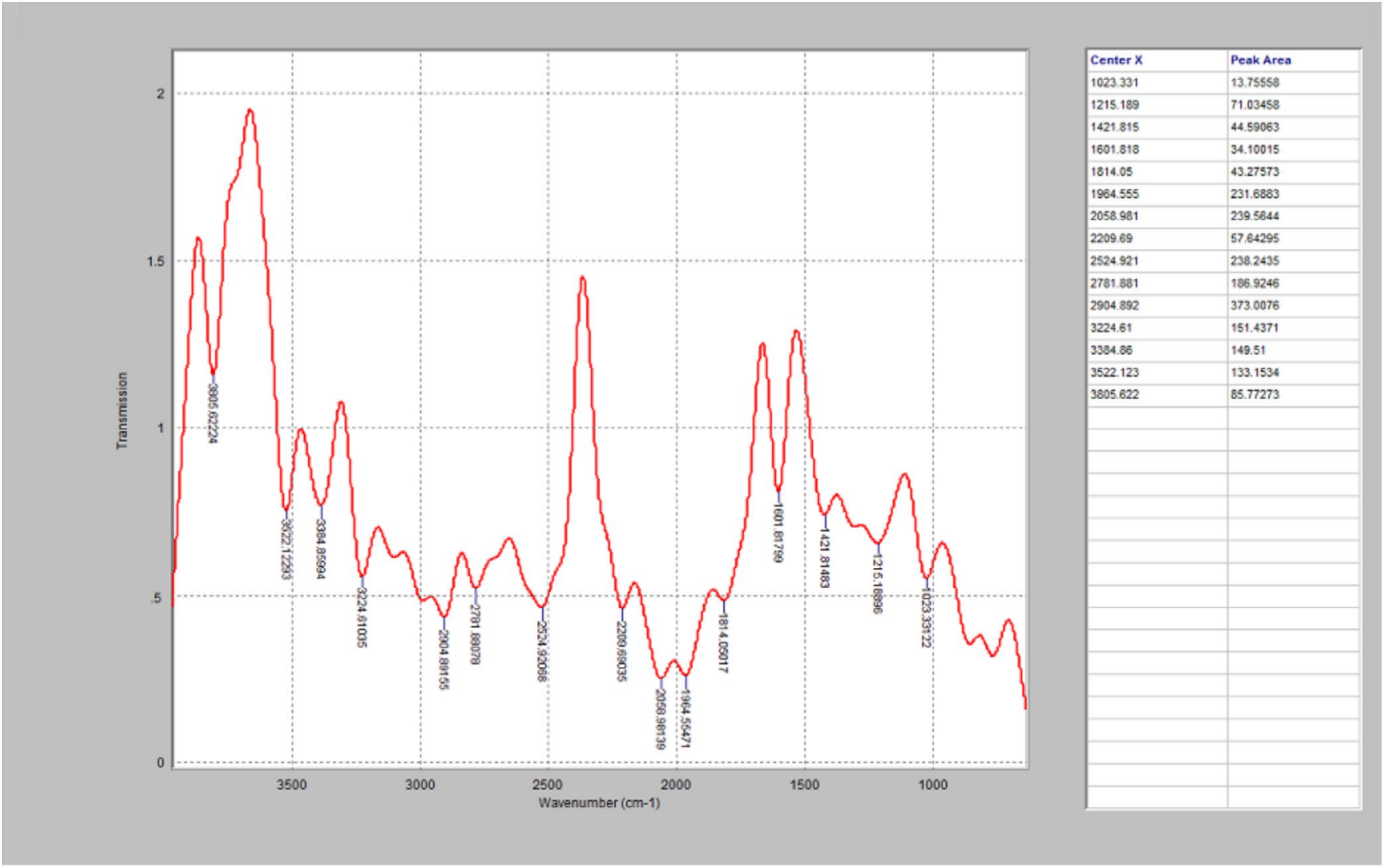
The FTIR diagram of the fresh untreated palm oil.

**FIGURE 3:**
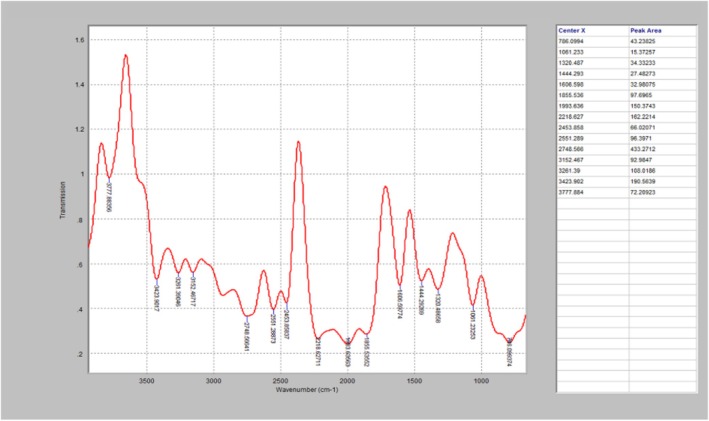
The FTIR diagram of PO blended with 4% hybridized EOs and heated for 150 min.

**FIGURE 4:**
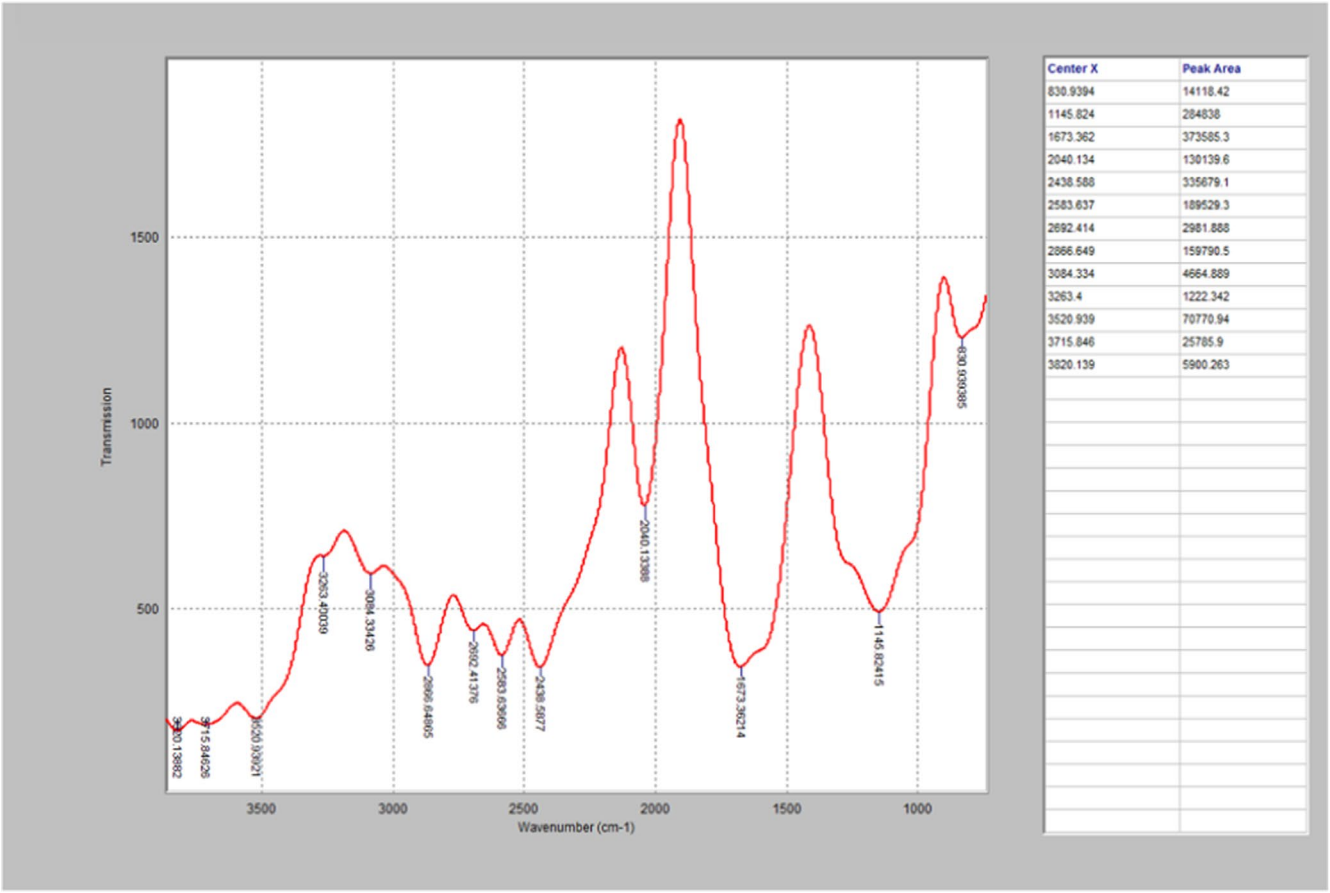
FTIR profile of the untreated palm and heated for 150 min.

The FTIR diagrams presented in Figures 2, 3, 4, further affirmed the effect of high temperature on the PO, as well as the degradation‐inhibition capability of the essential oil. As seen in Figure 2 (control), it has strong peaks at 2920 and 2850 cm^−1^, indicating the presence of intact aliphatic compounds such as CH_2_ and CH_3_, which established that the PO has undergone very slight oxidative alterations. Similarly, Figure 3 which is the FTIR analysis of one of the treated PO heated for 150 min, revealed that the PO has weaker peaks at 2920 and 2850 cm^−1^, and a broader O–H band. This indicates a slight degradation of hydrocarbon chains and the formation of oxidation products, which can be linked to thermal oxidation. The untreated PO (Figure 4) displayed the maximum thermal degradation, as its spectrum displays a substantial O–H stretching band and peak. This can be attributed to the presence of oxidation derivatives in the oil matrix.

According to David et al. (2023), curcumin has radical scavenging potential, as well as the ability to retard FTIR oxidation peaks. The degradation behaviors of oil are characterized by the FTIR spectral profile, which reflects the levels of lipid oxidation and polymerization reactions, which are caused by prolonged exposure to high temperatures (Wang et al. 2023). Expended heating decreased rapidly degrade the unsaturated fatty acids of palm oil (Egbung et al. 2024). The spectral profiles from the FTIR analysis established that heating caused significant degradation of the triglyceride structure, while the essential oils were able to inhibit both its breakdown, and the formation of polar oxidation materials. This is similar to the findings of Zhang et al. (2024), which reported that the chemical structures of oils are prone to deformation during thermal processing. Jointly, the results obtained from the FTIR spectra, validated the effects of heat processing and natural additives (essential oils) on the chemical integrity of palm oil, and the rapid thermal deterioration of PO's chemical properties in the UPO, tends to result in the formation of toxic oxidative compounds within the palm oil. Notably, the treated PO specimens exhibited a lower decrease in the C=C stretching and lower carbonyl absorption intensities, which can be attributed to actions against oxidation initiated by the antioxidants.

### 3.5 Modeling and Optimization of the Palm Oil Quality During Heating

The modeling and optimization of the palm oil thermal stability, with respect to the essential oils treatment, are presented in Tables 4, 5, 6, Tables S2–S4. Specifically, Table 4 shows the optimal results obtained from the optimization program, while Table S2 present the ANOVA for the Model Terms used in Optimization. Tables S3–S4 displayed the following –model section process for each response used for optimization and model equation for responses used for optimization, respectively. The ANOVA results depicted that both TUO (A), GO (B), BPO (C), and time (D) significantly influenced peroxide value, free fatty acid value, iodine value, carotenoids, tocopherols, viscosity, oxidative stability index, breakdown voltage, flash point (*p* < 0.05). This affirmed that the models consistently clarify the data variability, and they are significant. Notably, the ANOVA results have revealed that, heating duration and EO–PO collaborations serve essential functions, and the models developed were robust for the optimization task. Regarding the model section process for each response used in optimization results (Table S3), it was noted that the adjusted *R* ^2^ and predicted *R* ^2^ values were in close proximity. This validates the predictive capability of the models created deprived of over‐fitting. Specifically, the models designated ‘Suggested’ were used, as they have the superlative balance between precision and parsimony. Table S4 presents the values of the mathematical models created for each of the responses investigated in this study. The results revealed elevated values for *R* ^2^, adjusted *R* ^2^, and predicted *R* ^2^. As seen in the result most of the responses *R* ^2^ values were greater than 0.950 (close to 1), with smaller coefficient of variation values (< 5.00). The results reveal that, hybridizations and heating duration substantially affect the palm oil quality. The treated samples with higher volume of TUO showed the maximum desirability scores, which are an indication of a proportionate profile among the evaluated parameters. This affirmed the antioxidant effectiveness of curcumin compound. Furthermore, the Runs 21–26 samples recorded the minimum desirability scores, suggesting lower anti‐thermal degradation potential the BPO. This research results highlights the vital roles played by antioxidants in preserving the oil's quality during extended heating durations (Athanasiadis, Chatzimitakos, et al. 2024; Athanasiadis, Kalompatsios, et al. 2024; Yildiz et al. 2025).

**TABLE 4:** Optimum solutions obtained for the optimization.

S/N	TUO	GO	BPO	Time (minutes)	PV	FFA	IV	Carotenoids (mg/kg)	Tocopherols (mg/kg)	KV	OSI (h)	BDV (kV)	FP (°C)	Desirability
1	4.00	0.00	0.00	1.19	3.61	2.42	65.44	748.66	822.68	32.65	46.94	38.02	304.06	0.95
2	2.24	1.76	0.00	0.00	3.61	2.45	65.39	747.51	824.16	32.80	47.02	37.52	304.72	0.95
3	1.92	2.08	0.00	0.00	3.61	2.45	65.39	747.51	824.33	32.80	47.02	37.40	304.72	0.94
4	0.00	4.00	0.00	0.00	3.61	2.41	65.39	747.51	824.67	32.80	47.02	36.69	304.72	0.94
5	4.00	0.00	0.00	31.71	3.72	2.43	65.44	747.74	814.79	28.99	44.29	33.95	291.20	0.88
6	3.90	0.10	0.00	31.71	3.72	2.45	65.44	747.74	812.99	28.99	44.29	33.89	291.19	0.88
7	2.22	1.02	0.75	32.75	3.72	2.41	65.40	746.81	847.06	28.87	44.17	32.35	290.86	0.87
8	2.15	1.08	0.76	32.64	3.72	2.41	65.40	746.91	848.29	28.88	44.19	32.31	290.90	0.87
9	2.08	1.16	0.76	32.56	3.72	2.41	65.41	746.98	849.50	28.89	44.20	32.27	290.92	0.87
10	2.43	0.89	0.68	33.19	3.72	2.41	65.38	746.41	843.14	28.82	44.12	32.45	290.73	0.87
11	1.78	1.38	0.84	32.29	3.72	2.41	65.42	747.22	853.15	28.92	44.23	32.09	291.01	0.87
12	2.70	0.75	0.55	32.35	3.72	2.49	65.41	747.17	825.38	28.92	44.22	32.79	290.99	0.87
13	1.64	1.65	0.71	32.52	3.72	2.41	65.41	747.02	852.56	28.90	44.20	32.02	290.94	0.87
14	1.58	1.66	0.76	32.41	3.72	2.41	65.41	747.12	853.33	28.91	44.21	31.97	290.97	0.87
15	1.44	1.49	1.07	32.06	3.72	2.41	65.43	747.43	856.17	28.95	44.25	31.80	291.08	0.87
16	1.29	1.87	0.84	32.39	3.72	2.41	65.41	747.13	853.62	28.91	44.21	31.76	290.98	0.87
17	1.35	1.95	0.69	32.72	3.72	2.41	65.40	746.84	851.58	28.87	44.18	31.81	290.87	0.87
18	1.17	1.92	0.91	32.37	3.72	2.41	65.41	747.15	853.68	28.91	44.22	31.65	290.98	0.87
19	3.54	0.42	0.04	31.71	3.72	2.52	65.44	747.74	807.44	28.99	44.29	33.64	291.20	0.87
20	1.14	2.25	0.61	33.33	3.72	2.41	65.37	746.28	848.00	28.80	44.11	31.62	290.68	0.87
21	0.29	3.36	0.34	31.72	3.72	2.60	65.44	747.72	793.29	28.99	44.29	31.43	291.19	0.84
22	2.00	2.00	0.00	31.71	3.72	2.69	65.44	747.74	788.27	28.99	44.29	32.67	291.20	0.84
23	0.00	4.00	0.00	31.71	3.72	2.58	65.44	747.74	785.08	28.99	44.29	31.39	291.20	0.83
24	0.63	3.37	0.00	31.71	3.72	2.65	65.44	747.74	783.57	28.99	44.29	31.79	291.20	0.83
25	1.03	2.97	0.00	31.71	3.72	2.68	65.44	747.74	783.80	28.99	44.29	32.05	291.20	0.83
26	0.00	0.00	4.00	31.71	3.72	2.68	65.44	747.74	803.44	28.99	44.29	29.68	291.20	0.82

Table 5 shows that the specific hybridization of TUO, GO, and BPO at low concentrations substantially diminished PV and FFA levels in the PO, signifying an effective ability to resist oxidation reactions and hydrolytic depreciation. Likewise, the model revealed that the levels of carotenoid and tocopherol in the PO significantly contribute to the stability of OSI and BDV recorded in the PO during heating, which aligned to the findings documented by Čulina et al. (2025). Generally, the information provided by Table 4 affirmed that the optimization of the various treatments can serve as a sustainable blending material for vegetable oil. This will enhance the PO's nutritional, pharmaceutical, and engineering applications. Contrary to earlier scientific investigations, this research's findings have established that banana peels, which are an agricultural waste material, can be harnessed to produce an essential oil, which can be used in the thermal stabilization of vegetable oils, comparable to other organic materials.

**TABLE 5:** Constraints placed on the optimization processes.

Name	Goal	Lower limit	Upper limit	Lower weight	Upper weight	Imp
Independent variables						
A: Turmeric oil	IIR	0	4	1	1	3
B: Ginger oil	IIR	0	4	1	1	3
C: Banana peels oil	IIR	0	4	1	1	3
D: Time	IIR	0	150	1	1	3
Responses						
Peroxide value	Minimize	3.48	4.39	1	1	3
Free fatty acid value	Minimize	2.41	4.25	1	1	3
Iodine value	IIR	40.92	65.44	1	1	3
Carotenoids	Maximize	518.04	752.49	1	1	3
Tocopherols	Maximize	542.93	825.27	1	1	3
Kinematic viscosity	IIR	14.03	35.34	1	1	3
OSI	Maximize	17.34	50.63	1	1	3
BDV	Maximize	13.77	37.94	1	1	3
Flash point	IIR	235.84	311.34	1	1	3

Abbreviations: IIR, is in range; Imp, importance.

**FIGURE 5:**
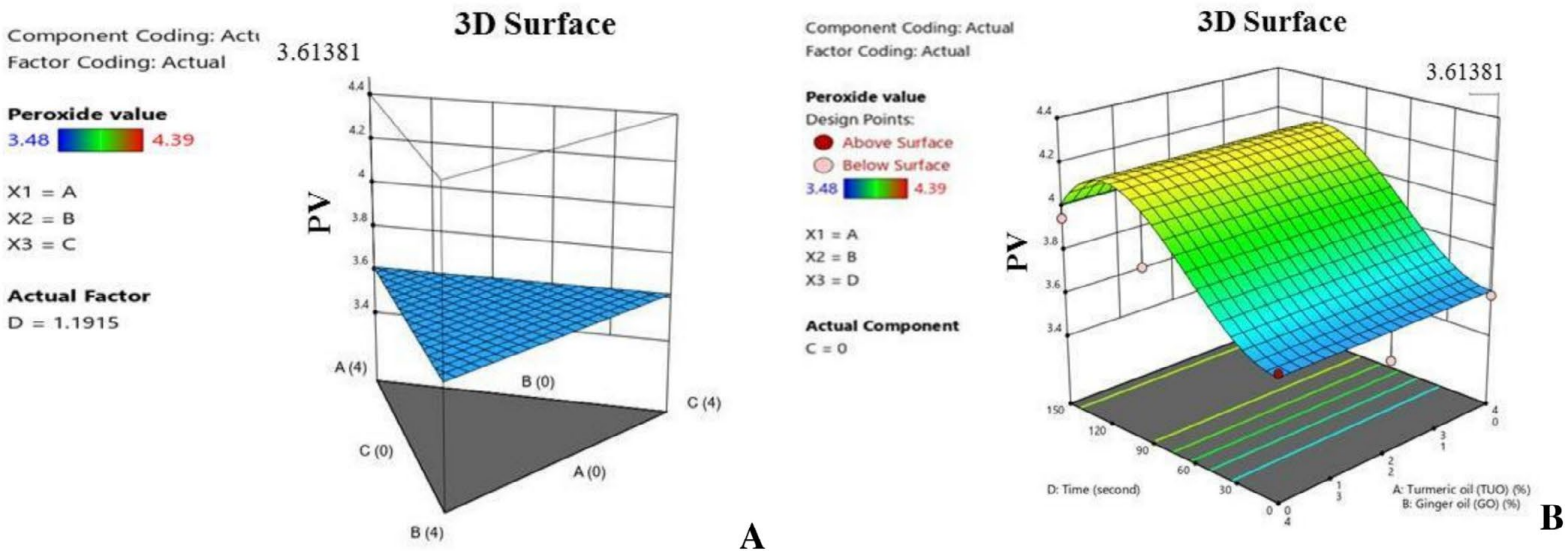
(A) Typical 3D graph of the effects of the mixtures on peroxide value. (B) Typical 3D graph of the effects of the mixtures and time on peroxide value.

**FIGURE 6:**
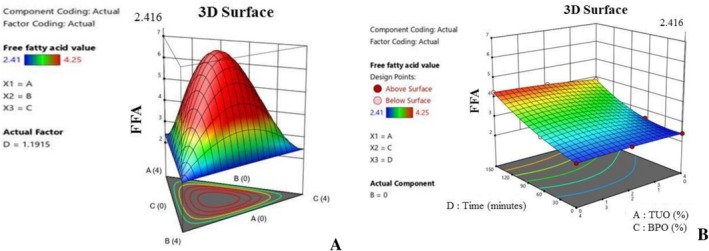
(A) 3D graph of the treatment effects on FFA value. (B) 3D graph of the effects of the additives and time on free fatty acid value.

**FIGURE 7:**
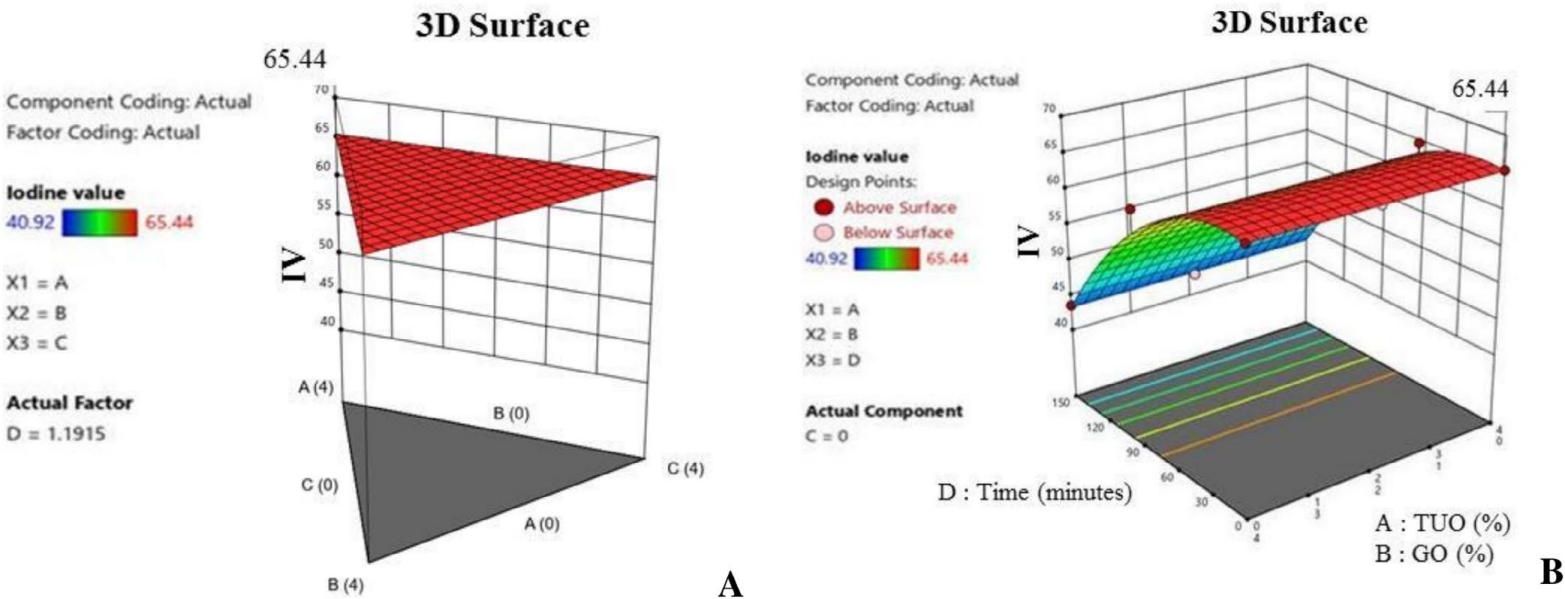
(A) Typical 3D graph of the effects of the mixtures on iodine value. (B) Typical 3D graph of the effects of the mixtures and time on iodine value.

**FIGURE 8:**
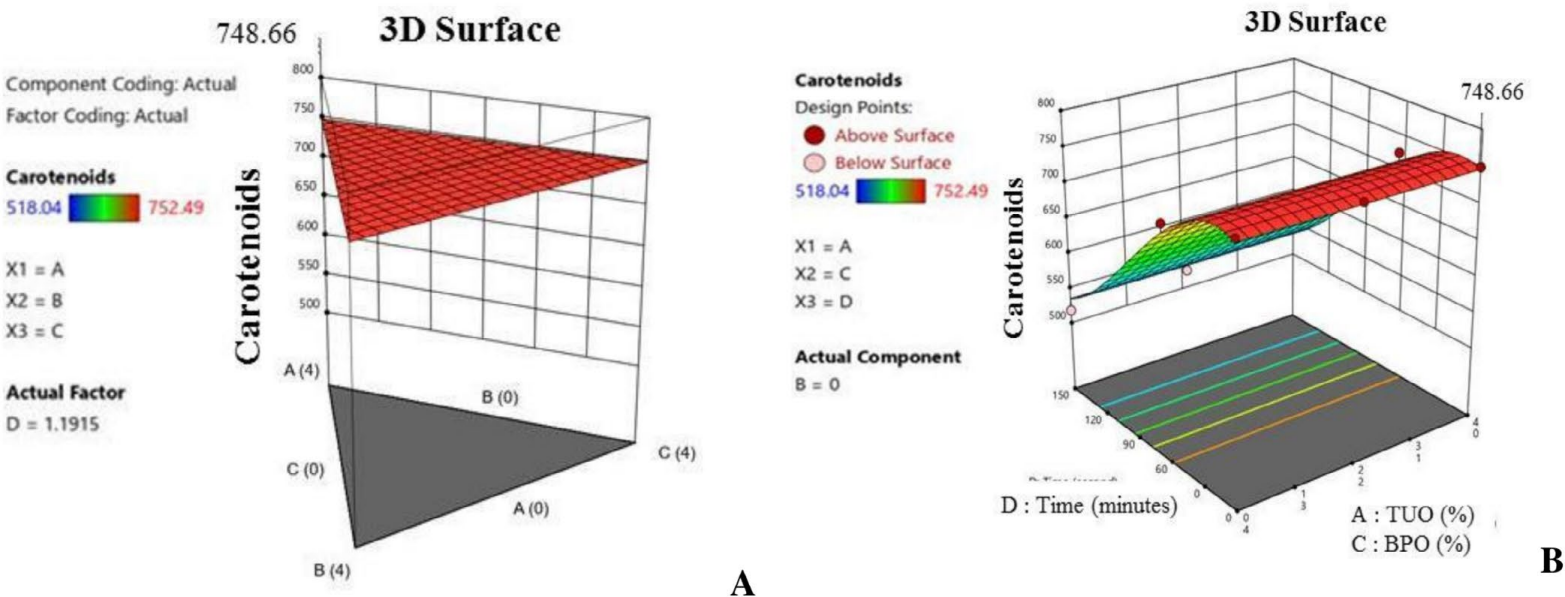
(A) 3D graph of the effects of treatment on carotenoids value. (B) 3D graph of the effects of treatment and time on carotenoids value.

**FIGURE 9:**
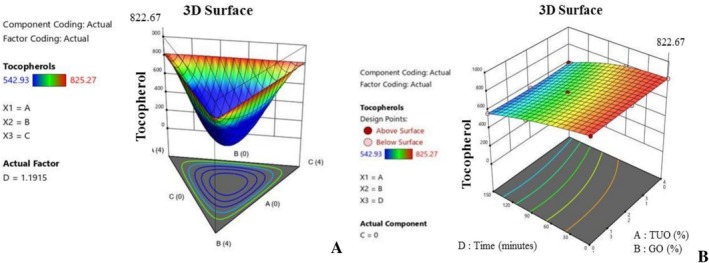
(A) The effects of treatment on tocopherols value. (B) The effects of treatment and time on tocopherols value.

**FIGURE 10:**
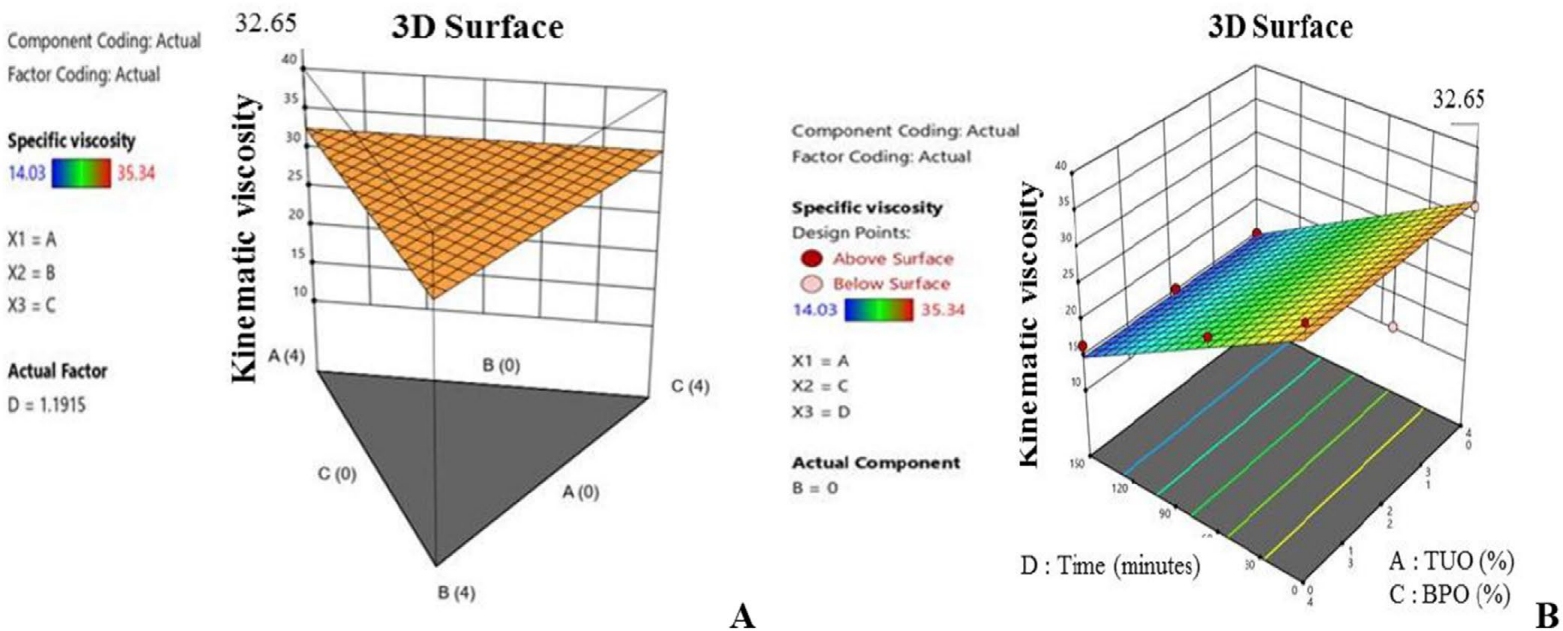
(A) Typical 3D graph of the effects of the mixtures on specific viscosity value. (B) Typical 3D graph of the effects of the mixtures and time on specific viscosity value.

**FIGURE 11:**
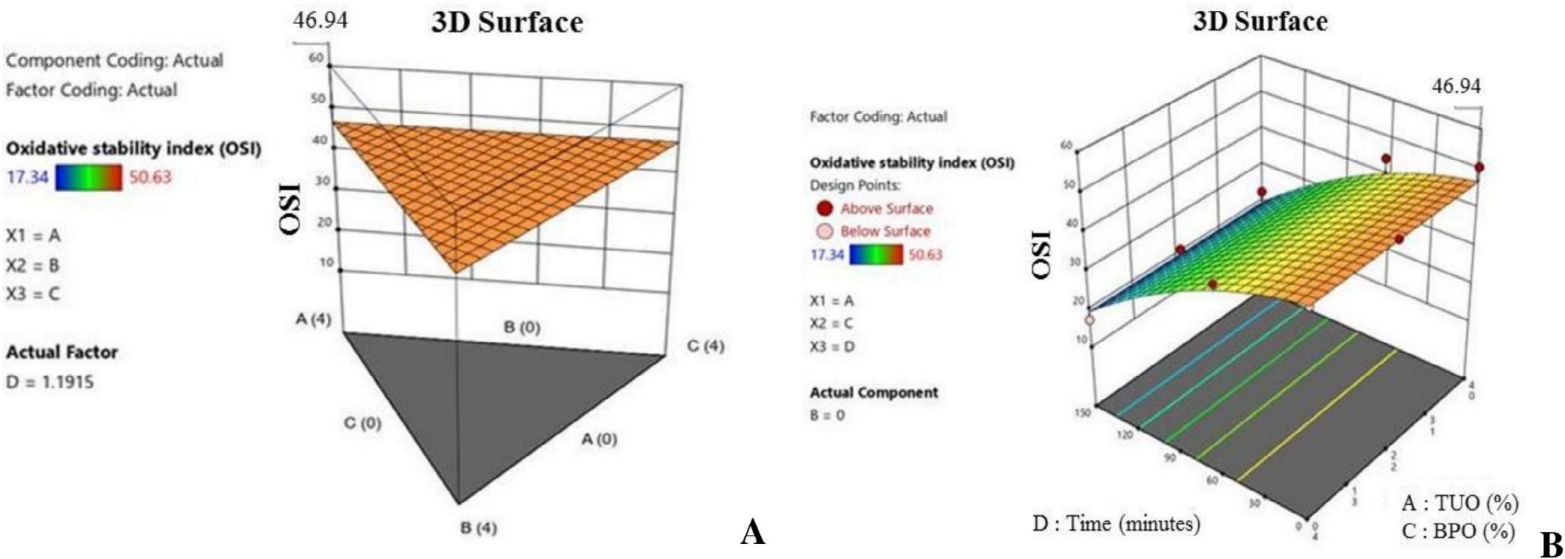
(A) Typical 3D graph of the effects of the mixtures on oxidative stability index (OSI) value. (B) Typical 3D graph of the effects of the mixtures and time on oxidative stability index (OSI) value.

**FIGURE 12:**
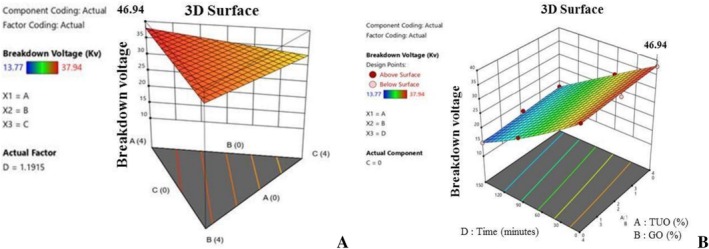
(A) 3D graph of the effects of the mixtures on breakdown voltage value. (B) 3D graph of the effects of the mixtures and time on breakdown voltage value.

**FIGURE 13:**
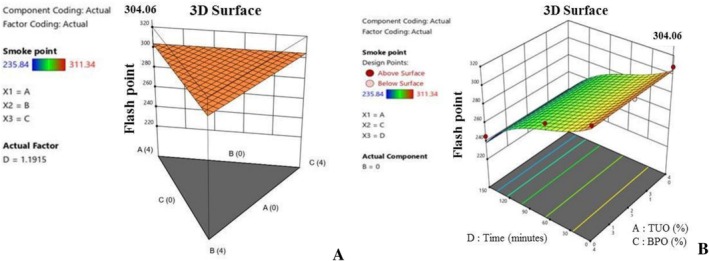
(A) The 3D graph of the effects of the treatment on flash point value. (B) The 3D graph of the effects of the treatment and time on flash point value.

Figures 5, 6, 7, 8, 9, 10, 11, 12, 13 show the interactive effects of the turmeric oil, ginger oil, banana peel oil hybridization, and heating duration on the palm oil's essential mechanical properties and bioactive compounds. The plots in Figure 5 show the influence of the treatment composition and heating duration, on the PO peroxide value. Oil peroxide value is among the essential indicators, which are used to evaluate oil's quality and oxidation level (Geng et al. 2023). Explicitly, Figure 5b revealed that the PO blends having greater TUO content maintained lower PV formation during the heating process, affirming the antioxidant effect of the EO in retarding the palm oil oxidation during prolong heating. This observation aligns with Gulcin (2025), who stated that antioxidants‐rich additives can potentially inhibit peroxide formation during high temperatures, by thwarting reactions that lead to harmful peroxide production.

Furthermore, Figure 6a,b show the influence of the additives on the palm oil FFA during heating. Notably, the blending affects the hydrolytic rancidity of the PO, and basically, Figure 6a shows that the hybridized mixtures with greater TUO and GO percentages have a higher ability to subdue FFA production during the heating operation. Free fatty acid is a critical factor in oil, as elevated values tend to lead to poor quality oil with health complications, such as cardiovascular diseases, inflammatory conditions, respiratory issues, liver disorder, and malabsorption problems (Uguru et al. 2023; Uçar et al. 2024). The higher FFA values recorded in the PO blended with lower antioxidants concentration is an indication that the PO has undergone a hydrolysis reaction. This results in the massive degradation of the oil's triglycerides into fatty acids and other relevant compounds. Additionally, the 3D plots (Figure 7a,b) show that oil fortification helps to preserve the IV content of the PO, and the higher concentration treatments tend to yield better results. Figure 8b established that the TUO and GO caused stability in the palm oil iodine content during heating, which could be attributed to these treatments' bioactive compounds, which retards the oxidation of the unsaturated fatty acids (Kaseke et al. 2020). It was noted that the hybridization helps to stabilize (suppressed) the depreciation of the iodine content during heating. Iodine content of the oil has numerous nutritional and medical benefits, such as anti‐inflammatory effects, high cholesterol management, and improvement of brain functions (Oboulbiga et al. 2023). Figure 8 shows the plot of the effect of the blending and heating time on the carotenoids content of the palm oil. Notably, Figure 8a,b indicate that the TUO and BPO substantially boost the carotenoids concentration, as well as offering greater resistance to the carotenoids during extended heating. This demonstrated the protective antioxidant capacity of the treatments, as they were able to retard carotenoid degradation during high temperatures. According to Gharby et al. (2025), a good natural additive should be able to hinder carotenoids and other essential medicinal properties of the oils during exposure to high temperatures, UV‐light, and prolonged storage.

Likewise, Figure 9 depicts the vitamin E content of the PO, which was optimized through the fortification with a mixture of TUO and GO, which is a situation that can be linked to the high antioxidant profiles of these two essential oils. Figure 9a established the collaborative relationship between the blending agents at precise blending ratios, which will result in enhanced tocopherol preservation during exposure to high temperatures. Similarly, Figure 9b reveals that the vitamin E level of the PO was susceptible to oxidation due to prolonged heating, and this condition can be linked to oxidative degradation. However, the presence of the hybridized additives in the palm oil was able to inhibit the oxidation. Precisely, treatments containing greater proportions of TUO and GO achieved maximum oxidation inhibition rates, and maintained higher tocopherol levels (Shahid et al. 2018; Uguru et al. 2023). Figure 10 shows that the PO kinematic viscosity value was substantially influenced by treatments' chemical composition, with BPO having the highest tendency to increase the PO viscosity. In Figure 10a, it was observed that the PO viscosity slightly increased when the oil contains higher percentages of BPO, which can be linked to the fatty acid structure of the BPO. Additionally, the interaction of TUO, GO, and BPO in the mixture yields mixed results, as some blends tend to increase the specific viscosity of the PO during heating, while others tend to decrease it during prolonged heating (Figure 10b). This behavioral pattern of the PO, with respect to the banana peel oil, could be associated with the anti‐thermal degradation ability of the bioactive compounds. During an investigation into oil preservation, Yildiz et al. (2025) noted that materials rich in antioxidants have the potential to alleviate the consequences of oils' decomposition.

Figure 11a,b show the diagrams for the 3D response surface plots, of the influence of plant‐derived essential oil (GEO) and time on the PO oxidative stability index (OSI). The figures affirmed the collaborative influence hybridization and the heating period on the oxidative behavior of the enriched palm oil samples. This signifies natural additives (stabilizer) effectiveness in enhancing edible oils' functional quality during prolonged heating. It was noted that the OSI value tends to increase with an increment in TUO and BPO concentrations, which depicted that the plant‐derived antioxidants had a positive impact on the PO's resistance to oxidative degradation (Figure 11a). The increment in the OSI can be linked to the high antioxidant concentration content of the several treatments used in this research. According to Hassanpour and Doroudi (2023), Phenolics and flavonoids have a high potential to stabilize oil quality by inhibiting lipid peroxidation. Additionally, Figure 11b illustrates the palm oil samples fortified with a greater TUO volume, significantly exhibiting higher inhibition of OSI degradation. This further affirms the protective influence of fortification agents on the oil quality. This is an indication that blending PO with the appropriate plant‐based antioxidants concentrations can efficiently preserve its storability and thermal stability, especially when the EOs are applied at optimized concentrations.

Additionally, Figure 12 presents the 3D graphical analysis of the impact of the treatment agents on the palm oil's BDV. It was observed that the TUO improves the oil's dielectric properties, as well as stabilising and retarding the degradation of the fortified PO's electrical breakdown voltage. Similarly, Figure 13a,b illustrate the flash point of the enriched PO samples, with respect to the blending concentrations. The figures revealed that the oil flash point was significantly impacted by the TUO and GO inclusion, resulting in an improvement in the palm oil's flash point, which will enhance the lubricating, cooling, and cooking performance of the blended PO. Generally, the optimisation results have revealed that the higher bioactive compounds presence in the blended PO has immensely helped in creating oxidative and thermal stability of the palm oil, hence retarding the production of FFA and PV during heating, resulting in better thermal resistance, and enhanced nutritional, medical, and engineering applications.

Finally, Table 6 presents the results of the confirmation test conducted to validate the optimization model used in this study. In the validation test, 4% of TUO was used to blend the PO, and the treated PO was heated for 150 min. The outcomes highlighted that the values obtained for all the parameters evaluated were within the acceptable range. Basically, the lower PV and FFA values recorded during the authentication test are an indication that lesser oxidation reaction and oil hydrolysis occurred when the treated PO was subjected to high temperature. Table 6 revealed that the predicted PV, FFA, OSI values were 4.02 meq O_2_/kg, 3.89 mg KOH/g, and 22.04 h, respectively, which was observed to fall within the 95% prediction interval. This indicates that the developed RSM model has perfect predictive accuracy, which is statistically trustworthy in the prediction of palm‐oil anti‐oxidation effect. Notably, the carotenoids and tocopherol contents in the enriched PO were still relatively high, regardless of the extended heating duration and elevated temperature, which is evidence of the powerful antioxidant capacity of the TUO (Orellana‐Paucar 2024). Moreover, the high OSI value (19.52 h) is additional confirmation of the oxidative resilience of the treated PO during high temperature processing or applications. According to Singh et al. (2025), curcumin, gallic acid, and other essential compounds present in essential oils play pivotal roles in reducing the oxidation level of palm oil, thereby increasing its integrity, functionality, and storability. Interestingly, the validation findings have affirmed that the forecasted and real‐time values aligned perfectly since the standard errors were of low values.

**TABLE 6:** Optimization model validation.

Response	Predicted Mean	Predicted Median	StdDev	*n*	SE Pred	95% PI low	Observation	95% PI high
PV	4.02	4.02	0.13	1	0.14	3.72	4.02	4.31
FFA	3.87	3.87	0.02	1	0.02	3.82	3.89	3.92
Iodine value	43.33	43.33	1.49	1	1.60	40.03	41.38	46.62
Carotenoids	535.36	535.36	19.90	1	21.47	491.05	550.26	579.67
Tocopherols	588.29	588.29	2.17	1	3.07	581.03	573.91	595.56
KV	14.81	14.81	2.01	1	2.11	10.47	14.21	19.15
OSI	19.52	19.52	2.14	1	2.30	14.79	22.04	24.25
BDV	17.86	17.86	1.09	1	1.41	14.90	16.48	20.81
Flash point	238.58	238.58	4.99	1	5.38	227.47	231.65	249.68

*Note:* The conditions under which the predicted results were obtained were: Turmeric oil (TUO) = 4.0% v/v, Ginger oil (GO) = 0% v/v, Banana peels oil (BPO) = 0% v/v, Time = 150 min.

Remarkably, the model validation results indicated that the RSM‐predicted optima are within the 95% forecasting intervals, representing a novelty compared to earlier research, which occasionally authenticates predicted antioxidant activities. Hence, the TUO and probably the GO and BPO can demonstrate strong efficiency in stabilizing the enriched PO exposed to extended high temperatures. Overall, these results highlight the importance of controlling the oil's oxidation by‐product (FFA and PV), mainly by incorporating natural antioxidants on the oil during applications that involve high temperatures, in order to preserve and stabilize the oil's quality. Therefore, the enriched PO samples tend to have a higher ability to preserve substantial pharmaceutical quality, dietary reliability, and functional integrity, which are pivotal in engineering, nutritional, and medical applications.

### 3.6 Limitation of the Study

Some critical factors, such as sensory evaluation, moisture content analysis, and smoke point, were not analyzed in this study. We recommend that further studies should look at these vital aspects. Also, the use of *n*‐hexane in essential oil extraction is a serious limitation, primarily due to public health safety concerns associated with this synthetic compound.

## 4 Conclusion

This research was conducted to evaluate the effectiveness of low volumes of essential oils (EOs), turmeric oil (TUO), ginger oil (GO), and banana peel oil (BPO) in inhibiting thermal degradation of palm oil (PO). The results established that the EOs blended with palm oil samples established a greater ability to maintain the oil's major nutritional and physicochemical properties during heating, which are significant in the food industry and other industrial applications where oil is exposed to extended high temperatures. Furthermore, the dielectric parameter investigated (breakdown voltage) supported the antioxidant effects of the EOs and the structural stability of enriched PO specimens when exposed to prolonged heating. The hybridization outcomes have confirmed that the tactical fortification of palm oil with essential oils can be successfully harnessed through optimization to attain specific application goals. Notably, the FTIR profiles results further confirmed the anti‐thermal oxidation effectiveness of the plant‐based oils. This study's results have shown the prospect of using plant‐based additives as natural antioxidants to preserve the bioengineering properties of palm oil for medical, domestic, and industrial applications. Based on the findings of this study, it is recommended that further research be conducted with a longer heating period, as well as the moisture content analysis of the palm oil, to evaluate real‐time frying and industrial applications.

## Author Contributions

**Manal A. Almalki:** methodology, conceptualization, funding acquisition, writing – review and editing. **H. Uguru:** methodology, conceptualization, funding acquisition, visualization. **O. Nyorere:** methodology, funding acquisition. **O. Eboibi:** methodology, conceptualization, funding acquisition. **O. Akpomedaye:** methodology, validation. **Nashi K. Alqahtani:** methodology, formal analysis, funding acquisition, writing – original draft. **Sarah Alharthi:** methodology, project administration. **Rokayya Sami:** methodology, writing – review and editing. **Norah E. Aljohani:** methodology, software. **Afnan M. Alnajeebi:** methodology, funding acquisition, investigation, resources, writing – review and editing. **Mahmoud Helal:** methodology, resources.

## Funding

The authors have nothing to report.

## Conflicts of Interest

The authors declare no conflicts of interest.

## Supporting information


**Table S1:** Calibration parameters for the phytochemicals.
**Table S2:** ANOVA for model terms used in optimization.
**Table S3:** Model section process for each response used for optimization.
**Table S4:** Model equation for responses used for optimization.

## Data Availability

The data of this study is available within the manuscript.
