# Supplementary material for: Enhancing the Multifunctional Qualities of Palm Oil With Plant‐Derived Essential Oils: An Integrated HPLC and RSM Approach
Source: Food Sci Nutr. 2026 Apr 15;14(4):e71741. doi: 10.1002/fsn3.71741 (PMC13081190; doi:10.1002/fsn3.71741)
Supplement: Supplementary file 1 — Table S1: Calibration parameters for the phytochemicals. Table S2: ANOVA for model terms used in optimization. Table S3: Model section process for each response used for optimization. Table S4: Model equation for responses used for optimization. [file FSN3-14-e71741-s001.docx]

**Supplementary Tables**

**Supplementary Table S1:** Calibration parameters for the phytochemicals

| **Parameter** | **Method** | **Absorbance wavelength (nm)** | **Reference Standard** | **Calibration Range** | **Regression Equation (y = ax + b)** | **R²** | **LOD** | **LOQ** |
| --- | --- | --- | --- | --- | --- | --- | --- | --- |
| α-Tocopherol | HPLC-UV | 292 | α-Tocopherol | 1–120 µg/mL | y = 24,382 x + 1,824 | 0.9991 | 0.14 µg/mL | 0.47 µg/mL |
| Total Phenolic Content | UV–Vis | 760 | GA | 2–250 mg GAE/g | y = 0.0061 x + 0.019 | 0.9990 | 0.89 mg GAE/g | 2.60 mg GAE/g |
| Total Flavonoid Content | UV–Vis | 415 | QUE | 5–290 mg QE/g | y = 0.0051 x + 0.019 | 0.9981 | 1.22 mg QE/g | 3.50 mg QE/g |
| Alkaloids | UV–Vis | 470 | Atropine | 4–150 mg AE/g | y = 0.0041 x + 0.024 | 0.9971 | 2.09 mg AE/g | 6.50 mg AE/g |
| Saponins | HPLC UV | 210 | Diosgenin | 10–300 mg DE/g | y = 0.0032 x + 0.028 | 0.9977 | 2.70 mg DE/g | 8.30 mg DE/g |
| Carotenoids | UV–Vis | 446 | β-Carotene | 1–40 µg/mg | y = 0.0192 x + 0.002 | 0.9981 | 0.07 µg/mg | 0.22 µg/mg |

QUE – Quercetin; LOQ - Limit of Quantification; LOD - Limit of Detection

The higher coefficient of determination value (R² ≥ 0.995) is an indication of the fittingness of the analytical approaches.

**Supplementary Tables: the parameters for the modeling**

**Table S2:** ANOVA for Model Terms used in Optimization

| Parameter | Source | Sum of  Squares | df | Mean  Square | F-value | p-value | Remark |
| --- | --- | --- | --- | --- | --- | --- | --- |
| Peroxide value | Model | 1.09 | 3 | 0.36 | 21.15 | 6.4E-07 | significant |
|  | D-Time | 0.29 | 1 | 0.29 | 16.60 | 4.4E-04 | significant |
|  | D² | 0.22 | 1 | 0.22 | 12.53 | 1.7E-03 | significant |
|  | D³ | 0.09 | 1 | 0.09 | 5.14 | 3.3E-02 | significant |
|  | Residual | 0.41 | 24 | 0.02 |  |  |  |
|  | Lack of Fit | 0.41 | 19 | 0.02 | 125.06 | 2.0E-05 | significant |
|  | Pure Error | 0.00 | 5 | 0.00 |  |  |  |
|  | Cor Total | 1.50 | 27 |  |  |  |  |
| Free fatty acid value | Model | 11.26 | 20 | 0.56 | 2206.45 | 2.0E-11 | significant |
|  | Linear Mixture | 0.44 | 2 | 0.22 | 855.57 | 4.3E-09 | significant |
|  | AB | 0.07 | 1 | 0.07 | 258.56 | 8.7E-07 | significant |
|  | AC | 0.00 | 1 | 0.00 | 14.05 | 7.2E-03 | significant |
|  | AD | 1.06 | 1 | 1.06 | 4155.29 | 5.7E-11 | significant |
|  | BC | 0.01 | 1 | 0.01 | 25.29 | 1.5E-03 | significant |
|  | BD | 1.61 | 1 | 1.61 | 6305.30 | 1.3E-11 | significant |
|  | CD | 1.68 | 1 | 1.68 | 6573.03 | 1.1E-11 | significant |
|  | ABC | 0.00 | 1 | 0.00 | 16.51 | 4.8E-03 | significant |
|  | ABD | 0.00 | 1 | 0.00 | 0.05 | 8.4E-01 | not significant |
|  | ACD | 0.00 | 1 | 0.00 | 0.71 | 4.3E-01 | not significant |
|  | BCD | 0.00 | 1 | 0.00 | 15.01 | 6.1E-03 | significant |
|  | AD² | 0.12 | 1 | 0.12 | 462.21 | 1.2E-07 | significant |
|  | BD² | 0.06 | 1 | 0.06 | 227.59 | 1.4E-06 | significant |
|  | CD² | 0.03 | 1 | 0.03 | 105.96 | 1.8E-05 | significant |
|  | ABCD | 0.03 | 1 | 0.03 | 107.70 | 1.7E-05 | significant |
|  | ABD² | 0.03 | 1 | 0.03 | 104.81 | 1.8E-05 | significant |
|  | ACD² | 0.00 | 1 | 0.00 | 9.38 | 1.8E-02 | significant |
|  | BCD² | 0.01 | 1 | 0.01 | 30.26 | 9.1E-04 | significant |
|  | ABCD² | 0.00 | 1 | 0.00 | 18.78 | 3.4E-03 | significant |
|  | Residual | 0.00 | 7 | 0.00 |  |  |  |
|  | Lack of Fit | 0.00 | 2 | 0.00 | 5.87 | 4.9E-02 | significant |
|  | Pure Error | 0.00 | 5 | 0.00 |  |  |  |
|  | Cor Total | 11.26 | 27 |  |  |  |  |
| Iodine value | Model | 2002.62 | 2 | 1001.31 | 451.26 | 2.4E-20 | significant |
|  | D-Time | 1732.55 | 1 | 1732.55 | 780.81 | 2.2E-20 | significant |
|  | D² | 270.78 | 1 | 270.78 | 122.03 | 4.1E-11 | significant |
|  | Residual | 55.47 | 25 | 2.22 |  |  |  |
|  | Lack of Fit | 55.47 | 20 | 2.77 | 208022.52 | 1.8E-13 | significant |
|  | Pure Error | 0.00 | 5 | 0.00 |  |  |  |
|  | Cor Total | 2058.10 | 27 |  |  |  |  |
| Carotenoids | Model | 188735.25 | 3 | 62911.75 | 158.80 | 5.8E-16 | significant |
|  | D-Time | 29184.87 | 1 | 29184.87 | 73.67 | 8.9E-09 | significant |
|  | D² | 6986.84 | 1 | 6986.84 | 17.64 | 3.2E-04 | significant |
|  | D³ | 2917.89 | 1 | 2917.89 | 7.37 | 1.2E-02 | significant |
|  | Residual | 9508.09 | 24 | 396.17 |  |  |  |
|  | Lack of Fit | 9293.67 | 19 | 489.14 | 11.41 | 6.7E-03 | significant |
|  | Pure Error | 214.42 | 5 | 42.88 |  |  |  |
|  | Cor Total | 198243.34 | 27 |  |  |  |  |
| Tocopherols | Model | 275419.13 | 20 | 13770.96 | 2915.63 | 7.7E-12 | significant |
|  | Linear Mixture | 7172.61 | 2 | 3586.30 | 759.30 | 6.5E-09 | significant |
|  | AB | 463.66 | 1 | 463.66 | 98.17 | 2.3E-05 | significant |
|  | AC | 254.91 | 1 | 254.91 | 53.97 | 1.6E-04 | significant |
|  | AD | 27602.90 | 1 | 27602.90 | 5844.18 | 1.7E-11 | significant |
|  | BC | 315.36 | 1 | 315.36 | 66.77 | 8.0E-05 | significant |
|  | BD | 33528.25 | 1 | 33528.25 | 7098.71 | 8.7E-12 | significant |
|  | CD | 37445.47 | 1 | 37445.47 | 7928.08 | 5.9E-12 | significant |
|  | ABC | 130.94 | 1 | 130.94 | 27.72 | 1.2E-03 | significant |
|  | ABD | 91.38 | 1 | 91.38 | 19.35 | 3.2E-03 | significant |
|  | ACD | 193.72 | 1 | 193.72 | 41.01 | 3.7E-04 | significant |
|  | BCD | 90.45 | 1 | 90.45 | 19.15 | 3.3E-03 | significant |
|  | AD² | 2393.05 | 1 | 2393.05 | 506.66 | 8.6E-08 | significant |
|  | BD² | 286.62 | 1 | 286.62 | 60.69 | 1.1E-04 | significant |
|  | CD² | 1896.61 | 1 | 1896.61 | 401.56 | 1.9E-07 | significant |
|  | ABCD | 290.23 | 1 | 290.23 | 61.45 | 1.0E-04 | significant |
|  | ABD² | 100.69 | 1 | 100.69 | 21.32 | 2.4E-03 | significant |
|  | ACD² | 189.45 | 1 | 189.45 | 40.11 | 3.9E-04 | significant |
|  | BCD² | 47.23 | 1 | 47.23 | 10.00 | 1.6E-02 | significant |
|  | ABCD² | 125.54 | 1 | 125.54 | 26.58 | 1.3E-03 | significant |
|  | Residual | 33.06 | 7 | 4.72 |  |  |  |
|  | Lack of Fit | 24.94 | 2 | 12.47 | 7.68 | 3.0E-02 | significant |
|  | Pure Error | 8.12 | 5 | 1.62 |  |  |  |
|  | Cor Total | 275452.20 | 27 |  |  |  |  |
| Specific viscosity | Model | 1151.67 | 1 | 1151.67 | 286.11 | 1.5E-15 | significant |
|  | D-Time | 1151.67 | 1 | 1151.67 | 286.11 | 1.5E-15 | significant |
|  | Residual | 104.66 | 26 | 4.03 |  |  |  |
|  | Lack of Fit | 104.16 | 21 | 4.96 | 49.87 | 1.9E-04 | significant |
|  | Pure Error | 0.50 | 5 | 0.10 |  |  |  |
|  | Cor Total | 1256.32 | 27 |  |  |  |  |
| Oxidative stability index (OSI) | Model | 2806.18 | 2 | 1403.09 | 306.75 | 2.6E-18 | significant |
|  | D-Time | 2690.39 | 1 | 2690.39 | 588.19 | 6.9E-19 | significant |
|  | D² | 116.37 | 1 | 116.37 | 25.44 | 3.3E-05 | significant |
|  | Residual | 114.35 | 25 | 4.57 |  |  |  |
|  | Lack of Fit | 114.32 | 20 | 5.72 | 883.90 | 1.5E-07 | significant |
|  | Pure Error | 0.03 | 5 | 0.01 |  |  |  |
|  | Cor Total | 2920.53 | 27 |  |  |  |  |
| Breakdown voltage | Model | 1592.40 | 8 | 199.05 | 168.32 | 5.6E-16 | significant |
|  | Linear Mixture | 78.61 | 2 | 39.30 | 33.24 | 6.2E-07 | significant |
|  | AD | 311.57 | 1 | 311.57 | 263.47 | 1.4E-12 | significant |
|  | BD | 372.16 | 1 | 372.16 | 314.70 | 2.8E-13 | significant |
|  | CD | 312.12 | 1 | 312.12 | 263.94 | 1.3E-12 | significant |
|  | AD² | 0.01 | 1 | 0.01 | 0.01 | 9.3E-01 | not significant |
|  | BD² | 1.26 | 1 | 1.26 | 1.06 | 3.2E-01 | not significant |
|  | CD² | 2.67 | 1 | 2.67 | 2.26 | 1.5E-01 | not significant |
|  | Residual | 22.47 | 19 | 1.18 |  |  |  |
|  | Lack of Fit | 22.27 | 14 | 1.59 | 39.92 | 3.6E-04 | significant |
|  | Pure Error | 0.20 | 5 | 0.04 |  |  |  |
|  | Cor Total | 1614.87 | 27 |  |  |  |  |
| Smoke point | Model | 14792.62 | 3 | 4930.87 | 198.19 | 4.6E-17 | significant |
|  | D-Time | 481.37 | 1 | 481.37 | 19.35 | 1.9E-04 | significant |
|  | D² | 333.25 | 1 | 333.25 | 13.39 | 1.2E-03 | significant |
|  | D³ | 148.72 | 1 | 148.72 | 5.98 | 2.2E-02 | significant |
|  | Residual | 597.10 | 24 | 24.88 |  |  |  |
|  | Lack of Fit | 595.35 | 19 | 31.33 | 89.26 | 4.6E-05 | significant |
|  | Pure Error | 1.76 | 5 | 0.35 |  |  |  |
|  | Cor Total | 15389.73 | 27 |  |  |  |  |
| A is Turmeric oil (TUO), B is Ginger oil (GO), C is Banana peels oil (BPO), D is Time | | | | | |  |  |

**Table S3:** Model Section Process for each Response used for Optimization

| Parameter | Mixture  Order | Process  Order | Mixture  p-value | Process  p-value | Lack of Fit  p-value | Adjusted  R² | Predicted  R² | Remark |
| --- | --- | --- | --- | --- | --- | --- | --- | --- |
| Peroxide value | M | M |  |  |  |  |  |  |
|  | M | L |  | 1.46E-05 | 6.33E-06 | 0.50243 | 0.43733 |  |
|  | M | Q |  | 0.0028472 | 1.40E-05 | 0.64009 | 0.58737 |  |
|  | M | C |  | 0.0326343 | 2.01E-05 | 0.69125 | 0.62247 | **Suggested** |
|  | L | M | 0.3461993 |  | 1.11E-06 | 0.00787 | -0.12515 |  |
|  | L | L | 0.3035352 | 0.0002309 | 6.56E-06 | 0.52384 | 0.26645 |  |
|  | L | Q | 0.1571157 | 0.008717 | 1.87E-05 | 0.69684 | 0.39423 |  |
|  | L | C | 0.0830928 | 0.0430644 | 3.73E-05 | 0.78050 | 0.47264 |  |
|  | Q | M | 0.5504188 |  | 9.66E-07 | -0.02657 | -0.29864 |  |
|  | Q | L | 0.4070343 | 0.0030883 | 5.78E-06 | 0.53583 | -0.41586 |  |
|  | Q | Q | 0.0573966 | 0.0066359 | 4.99E-05 | 0.83965 | -12.58610 |  |
|  | Q | C | 3.73E-05 | 4.99E-05 |  | 0.99689 |  | Aliased |
|  | SC | M | 0.4786978 |  | 8.93E-07 | -0.04945 | -0.42511 |  |
|  | SC | L | 0.1568517 | 0.0027647 | 7.20E-06 | 0.59287 | -0.68279 |  |
|  | SC | Q | 0.0008147 | 7.58E-05 | 0.002647341 | 0.97613 | -36.65995 |  |
|  | SC | C |  | 0.0026473 |  | 0.99689 |  | Aliased |
|  | C | M | 0.6841038 |  | 6.81E-07 | -0.12942 | -3.55100 |  |
|  | C | L | 2.61E-05 | 7.73E-07 | 0.002958383 | 0.97373 |  | **Suggested** |
|  | C | Q | 0.0026473 | 0.0029584 |  | 0.99689 |  | Aliased |
|  | C | C |  |  |  | 0.99689 |  | Aliased |
| Free fatty acid value | M | M |  |  |  |  |  |  |
|  | M | L |  | 1.91E-17 | 2.25E-06 | 0.93810 | 0.93176 |  |
|  | M | Q |  | 9.43E-05 | 9.46E-06 | 0.96543 | 0.96062 |  |
|  | M | C |  | 0.0125575 | 1.62E-05 | 0.97237 | 0.96645 |  |
|  | L | M | 0.6100192 |  | 1.93E-09 | -0.03813 | -0.25258 |  |
|  | L | L | 0.1130696 | 5.50E-15 | 3.11E-06 | 0.94717 | 0.92502 |  |
|  | L | Q | 0.0067346 | 5.37E-05 | 3.52E-05 | 0.98068 | 0.96981 |  |
|  | L | C | 0.0064912 | 0.028045 | 8.10E-05 | 0.98679 | 0.97108 |  |
|  | Q | M | 0.9737074 |  | 1.36E-09 | -0.16803 | -0.67415 |  |
|  | Q | L | 0.9744198 | 4.18E-10 | 1.38E-06 | 0.93225 | 0.67839 |  |
|  | Q | Q | 0.1121586 | 0.0003069 | 6.09E-05 | 0.98784 | -0.03114 |  |
|  | Q | C | 8.10E-05 | 6.09E-05 |  | 0.99974 |  | Aliased |
|  | SC | M | 0.7389623 |  | 1.20E-09 | -0.21704 | -0.78183 |  |
|  | SC | L | 0.5958313 | 9.12E-09 | 1.07E-06 | 0.92809 | 0.63029 |  |
|  | SC | Q | 1.88E-05 | 9.27E-08 | 0.048726533 | 0.99939 | 0.58177 | **Suggested** |
|  | SC | C |  | 0.0487265 |  | 0.99974 |  | Aliased |
|  | C | M | 0.7667526 |  | 8.71E-10 | -0.33476 | -3.19777 |  |
|  | C | L | 0.310671 | 1.09E-05 | 7.26E-07 | 0.93934 |  |  |
|  | C | Q | 0.0487265 | 7.26E-07 |  | 0.99974 |  | Aliased |
|  | C | C |  |  |  | 0.99974 |  | Aliased |
| Iodine value | M | M |  |  |  |  |  |  |
|  | M | L |  | 6.70E-12 | 2.40E-15 | 0.83538 | 0.81558 |  |
|  | M | Q |  | 4.14E-11 | 1.80E-13 | 0.97089 | 0.96583 | **Suggested** |
|  | M | C |  | 0.3450429 | 1.75E-13 | 0.97081 | 0.96444 |  |
|  | L | M | 0.7675698 |  | 2.26E-17 | -0.05739 | -0.28782 |  |
|  | L | L | 0.8429465 | 3.67E-09 | 1.71E-15 | 0.81701 | 0.69275 |  |
|  | L | Q | 0.3136108 | 1.19E-08 | 1.84E-13 | 0.97271 | 0.95227 |  |
|  | L | C | 0.1781083 | 0.1640772 | 2.32E-13 | 0.97623 | 0.94556 |  |
|  | Q | M | 0.9093351 |  | 1.65E-17 | -0.17291 | -0.71420 |  |
|  | Q | L | 0.9456682 | 5.66E-06 | 8.07E-16 | 0.77108 | -0.09602 |  |
|  | Q | Q | 0.0040039 | 8.57E-08 | 2.33E-12 | 0.99225 | 0.43806 |  |
|  | Q | C | 2.32E-13 | 2.33E-12 |  | 1.00000 |  | Aliased |
|  | SC | M | 0.6705576 |  | 1.46E-17 | -0.21796 | -0.79832 |  |
|  | SC | L | 0.9456175 | 5.67E-05 | 5.29E-16 | 0.74046 | -0.26829 |  |
|  | SC | Q | 0.0076466 | 9.34E-08 | 2.40E-11 | 0.99779 | -1.46303 | **Suggested** |
|  | SC | C |  | 2.40E-11 |  | 1.00000 |  | Aliased |
|  | C | M | 0.7502317 |  | 1.07E-17 | -0.33082 | -2.88176 |  |
|  | C | L | 0.3697132 | 0.0019966 | 3.06E-16 | 0.76669 |  |  |
|  | C | Q | 2.40E-11 | 3.06E-16 |  | 1.00000 |  | Aliased |
|  | C | C |  |  |  | 1.00000 |  | Aliased |
| Carotenoids | M | M |  |  |  |  |  |  |
|  | M | L |  | 1.14E-14 | 0.001536923 | 0.89897 | 0.88869 |  |
|  | M | Q |  | 0.0010252 | 0.003971774 | 0.93231 | 0.92430 |  |
|  | M | C |  | 0.0121141 | 0.006703523 | 0.94604 | 0.93254 | **Suggested** |
|  | L | M | 0.7358461 |  | 4.61E-06 | -0.05382 | -0.25700 |  |
|  | L | L | 0.5137745 | 7.51E-12 | 0.001351144 | 0.89644 | 0.86956 |  |
|  | L | Q | 0.5197215 | 0.0139552 | 0.003298956 | 0.93052 | 0.89420 |  |
|  | L | C | 0.7300317 | 0.1775188 | 0.004041311 | 0.93880 | 0.84034 |  |
|  | Q | M | 0.9821405 |  | 3.22E-06 | -0.18846 | -0.65364 |  |
|  | Q | L | 0.9782964 | 7.63E-08 | 0.000607273 | 0.86662 | 0.49439 |  |
|  | Q | Q | 0.7965676 | 0.1058268 | 0.001068988 | 0.91259 | -5.68117 |  |
|  | Q | C | 0.0040413 | 0.001069 |  | 0.99416 |  | Aliased |
|  | SC | M | 0.9567586 |  | 2.81E-06 | -0.24487 | -0.80231 |  |
|  | SC | L | 0.0940076 | 1.35E-07 | 0.000904595 | 0.89126 | 0.42412 |  |
|  | SC | Q | 0.0009709 | 0.0010277 | 0.051290096 | 0.98631 | -12.41166 | **Suggested** |
|  | SC | C |  | 0.0512901 |  | 0.99416 |  | Aliased |
|  | C | M | 0.7127005 |  | 2.11E-06 | -0.34864 | -3.36869 |  |
|  | C | L | 0.3323655 | 5.83E-05 | 0.000597202 | 0.90601 |  |  |
|  | C | Q | 0.0512901 | 0.0005972 |  | 0.99416 |  | Aliased |
|  | C | C |  |  |  | 0.99416 |  | Aliased |
| Tocopherols | M | M |  |  |  |  |  |  |
|  | M | L |  | 1.84E-19 | 1.68E-06 | 0.95667 | 0.95148 |  |
|  | M | Q |  | 1.41E-06 | 1.59E-05 | 0.98254 | 0.97979 |  |
|  | M | C |  | 0.0004046 | 5.30E-05 | 0.98931 | 0.98704 |  |
|  | L | M | 0.7190637 |  | 5.72E-10 | -0.05188 | -0.26007 |  |
|  | L | L | 0.3835325 | 4.61E-16 | 1.62E-06 | 0.95729 | 0.93461 |  |
|  | L | Q | 0.0164 | 1.84E-06 | 4.50E-05 | 0.98911 | 0.97175 |  |
|  | L | C | 0.0620974 | 0.0227837 | 0.000111037 | 0.99276 | 0.97830 |  |
|  | Q | M | 0.991421 |  | 3.97E-10 | -0.18983 | -0.68652 |  |
|  | Q | L | 0.984063 | 7.33E-11 | 6.98E-07 | 0.94459 | 0.71227 |  |
|  | Q | Q | 0.041145 | 1.45E-05 | 0.000147671 | 0.99470 | 0.85679 |  |
|  | Q | C | 0.000111 | 0.0001477 |  | 0.99984 |  | Aliased |
|  | SC | M | 0.5335801 |  | 3.62E-10 | -0.22315 | -0.77986 |  |
|  | SC | L | 0.4729836 | 1.74E-09 | 5.86E-07 | 0.94309 | 0.69861 |  |
|  | SC | Q | 0.0001283 | 7.93E-08 | 0.029897729 | 0.99954 | 0.51736 | **Suggested** |
|  | SC | C |  | 0.0298977 |  | 0.99984 |  | Aliased |
|  | C | M | 0.7820583 |  | 2.61E-10 | -0.34608 | -2.38373 |  |
|  | C | L | 0.4615679 | 7.58E-06 | 2.74E-07 | 0.94424 |  |  |
|  | C | Q | 0.0298977 | 2.74E-07 |  | 0.99984 |  | Aliased |
|  | C | C |  |  |  | 0.99984 |  | Aliased |
| Specific viscosity | M | M |  |  |  |  |  |  |
|  | M | L |  | 1.50E-15 | 0.000191699 | 0.91349 | 0.89845 | **Suggested** |
|  | M | Q |  | 0.4993578 | 0.000179384 | 0.91169 | 0.89043 |  |
|  | M | C |  | 0.1141879 | 0.000207216 | 0.91728 | 0.89571 |  |
|  | L | M | 0.8532953 |  | 3.64E-07 | -0.06638 | -0.28410 |  |
|  | L | L | 0.238198 | 4.11E-13 | 0.000213824 | 0.91960 | 0.89154 |  |
|  | L | Q | 0.417175 | 0.7226283 | 0.00016321 | 0.91304 | 0.82975 |  |
|  | L | C | 0.6340901 | 0.5050123 | 0.000135763 | 0.91040 | 0.79663 |  |
|  | Q | M | 0.9944325 |  | 2.52E-07 | -0.20765 | -0.72019 |  |
|  | Q | L | 0.1677077 | 2.72E-10 | 0.000285232 | 0.93364 | 0.84032 |  |
|  | Q | Q | 0.0212373 | 0.0493422 | 0.000792624 | 0.96388 | 0.06358 |  |
|  | Q | C | 0.0001358 | 0.0007926 |  | 0.99786 |  | Aliased |
|  | SC | M | 0.4420429 |  | 2.36E-07 | -0.22921 | -0.79706 |  |
|  | SC | L | 0.1011074 | 1.28E-09 | 0.000414585 | 0.94534 | 0.76533 |  |
|  | SC | Q | 0.0015287 | 0.0008268 | 0.027254859 | 0.99355 | -3.80492 |  |
|  | SC | C |  | 0.0272549 |  | 0.99786 |  | Aliased |
|  | C | M | 0.7513964 |  | 1.73E-07 | -0.34346 | -3.24980 |  |
|  | C | L | 7.65E-06 | 3.73E-11 | 0.30965547 | 0.99742 |  | **Suggested** |
|  | C | Q | 0.0272549 | 0.3096555 |  | 0.99786 |  | Aliased |
|  | C | C |  |  |  | 0.99786 |  | Aliased |
| Oxidative stability index (OSI) | M | M |  |  |  |  |  |  |
|  | M | L |  | 7.51E-16 | 2.95E-08 | 0.91796 | 0.90866 |  |
|  | M | Q |  | 3.33E-05 | 1.52E-07 | 0.95771 | 0.95079 | **Suggested** |
|  | M | C |  | 0.3796665 | 1.47E-07 | 0.95737 | 0.94783 |  |
|  | L | M | 0.6269667 |  | 5.08E-11 | -0.04041 | -0.27482 |  |
|  | L | L | 0.2097658 | 2.55E-13 | 3.42E-08 | 0.92489 | 0.89757 |  |
|  | L | Q | 0.0873818 | 0.0002762 | 2.51E-07 | 0.96722 | 0.93113 |  |
|  | L | C | 0.1383586 | 0.4475908 | 2.18E-07 | 0.96687 | 0.90035 |  |
|  | Q | M | 0.9601206 |  | 3.60E-11 | -0.16664 | -0.71310 |  |
|  | Q | L | 0.8514033 | 3.67E-09 | 1.85E-08 | 0.91093 | 0.58857 |  |
|  | Q | Q | 0.0548294 | 0.0004279 | 6.89E-07 | 0.98286 | -0.22561 |  |
|  | Q | C | 2.18E-07 | 6.89E-07 |  | 0.99994 |  | Aliased |
|  | SC | M | 0.6988079 |  | 3.19E-11 | -0.21330 | -0.80306 |  |
|  | SC | L | 0.8531186 | 8.74E-08 | 1.26E-08 | 0.90050 | 0.51903 |  |
|  | SC | Q | 0.0031226 | 1.68E-05 | 1.37E-05 | 0.99623 | -3.10238 | **Suggested** |
|  | SC | C |  | 1.37E-05 |  | 0.99994 |  | Aliased |
|  | C | M | 0.8205366 |  | 2.25E-11 | -0.34678 | -2.25687 |  |
|  | C | L | 0.2525789 | 2.84E-05 | 1.02E-08 | 0.92192 |  |  |
|  | C | Q | 1.37E-05 | 1.02E-08 |  | 0.99994 |  | Aliased |
|  | C | C |  |  |  | 0.99994 |  | Aliased |
| Breakdown voltage | M | M |  |  |  |  |  |  |
|  | M | L |  | 4.85E-19 | 4.93E-05 | 0.95332 | 0.94668 |  |
|  | M | Q |  | 0.7856717 | 4.43E-05 | 0.95160 | 0.94252 |  |
|  | M | C |  | 0.0057664 | 8.80E-05 | 0.96354 | 0.95496 |  |
|  | L | M | 0.5359175 |  | 2.17E-08 | -0.02743 | -0.23429 |  |
|  | L | L | 9.89E-05 | 1.23E-19 | 0.000385128 | 0.98027 | 0.97500 |  |
|  | L | Q | 0.0004666 | 0.4204717 | 0.0003554 | 0.98023 | 0.97466 |  |
|  | L | C | 2.11E-06 | 4.42E-05 | 0.006151509 | 0.99414 | 0.98628 | **Suggested** |
|  | Q | M | 0.9787117 |  | 1.52E-08 | -0.15756 | -0.63546 |  |
|  | Q | L | 0.8019345 | 7.99E-14 | 0.000222367 | 0.97712 | 0.95316 |  |
|  | Q | Q | 0.9838273 | 0.9343611 | 6.25E-05 | 0.96864 | -0.06720 |  |
|  | Q | C | 0.0061515 | 6.25E-05 |  | 0.99933 |  | Aliased |
|  | SC | M | 0.4966522 |  | 1.40E-08 | -0.18566 | -0.73156 |  |
|  | SC | L | 0.0001155 | 1.15E-15 | 0.003449267 | 0.99284 | 0.97752 |  |
|  | SC | Q | 0.0002876 | 0.0768622 | 0.007040326 | 0.99654 | -3.78775 |  |
|  | SC | C |  | 0.0070403 |  | 0.99933 |  | Aliased |
|  | C | M | 0.7819519 |  | 1.01E-08 | -0.30478 | -1.92032 |  |
|  | C | L | 0.0497268 | 1.25E-10 | 0.010257717 | 0.99661 |  | **Suggested** |
|  | C | Q | 0.0070403 | 0.0102577 |  | 0.99933 |  | Aliased |
|  | C | C |  |  |  | 0.99933 |  | Aliased |
| Smoke point | M | M |  |  |  |  |  |  |
|  | M | L |  | 1.71E-16 | 1.32E-05 | 0.92676 | 0.91958 |  |
|  | M | Q |  | 0.0024172 | 3.00E-05 | 0.94766 | 0.94131 |  |
|  | M | C |  | 0.0222054 | 4.63E-05 | 0.95635 | 0.94578 | **Suggested** |
|  | L | M | 0.8127725 |  | 1.64E-08 | -0.06224 | -0.28716 |  |
|  | L | L | 0.5129257 | 2.02E-13 | 1.16E-05 | 0.92494 | 0.90349 |  |
|  | L | Q | 0.4164619 | 0.0171107 | 2.73E-05 | 0.94848 | 0.93102 |  |
|  | L | C | 0.5070494 | 0.1530037 | 3.53E-05 | 0.95557 | 0.89979 |  |
|  | Q | M | 0.9996455 |  | 1.13E-08 | -0.20644 | -0.75988 |  |
|  | Q | L | 0.9573395 | 4.59E-09 | 5.35E-06 | 0.90525 | 0.75882 |  |
|  | Q | Q | 0.9609269 | 0.2453212 | 5.38E-06 | 0.92251 | -0.87024 |  |
|  | Q | C | 3.53E-05 | 5.38E-06 |  | 0.99938 |  | Aliased |
|  | SC | M | 0.8970926 |  | 9.82E-09 | -0.26285 | -0.85382 |  |
|  | SC | L | 0.0843924 | 1.04E-08 | 8.31E-06 | 0.92394 | 0.64138 |  |
|  | SC | Q | 0.0005646 | 0.0013462 | 0.000370308 | 0.98963 | -10.23063 | **Suggested** |
|  | SC | C |  | 0.0003703 |  | 0.99938 |  | Aliased |
|  | C | M | 0.950722 |  | 6.41E-09 | -0.44584 | -2.61611 |  |
|  | C | L | 0.0603876 | 1.28E-06 | 2.07E-05 | 0.96184 |  |  |
|  | C | Q | 0.0003703 | 2.07E-05 |  | 0.99938 |  | Aliased |
|  | C | C |  |  |  | 0.99938 |  | Aliased |
| M is mean, L is Linear, Q is Quadratic, SC is Special Cubic, C is Cubic | | | | | |  |  |  |
| Aliased is when model chosen is insufficient to fully separate the effects of all terms being investigated | | | | | | | |  |

**Table S4:** Model Equation for Responses used for Optimization

| **Parameter** | **Equation** | **Std. Dev.** | **Mean** | **C.V. %** | **R²** | **Adjusted R²** | **Predicted R²** | **Adeq Precision** |
| --- | --- | --- | --- | --- | --- | --- | --- | --- |
| Peroxide value | Peroxide value = 3.6137093242419 -6.4122641683795E-05 Time + 0.00012578964712259 Time² -7.1655189476299E-07 Time³ | 0.1311385 | 3.9078571 | 3.3557639 | 0.7255516 | 0.691246 | 0.622474 | 11.40859 |
| Free fatty acid value | Free fatty acid value = 0.60458967523392 Turmeric oil (TUO) + 0.60347011650841 Ginger oil (GO) + 0.60949741980696 Banana peels oil (BPO) + 0.0088469500558249 Turmeric oil (TUO) Ginger oil (GO) + 0.0095707361631388 Turmeric oil (TUO) Banana peels oil (BPO) -0.00050951054693455 Turmeric oil (TUO) Time + 0.0040603846036124 Ginger oil (GO) Banana peels oil (BPO) + 0.000891432648407 Ginger oil (GO) Time + 0.0016237626249762 Banana peels oil (BPO) Time + 1.9747257968953 Turmeric oil (TUO) Ginger oil (GO) Banana peels oil (BPO) + 0.0014323706361812 Turmeric oil (TUO) Ginger oil (GO) Time + 0.0017112944032259 Turmeric oil (TUO) Banana peels oil (BPO) Time + 0.00063598403280511 Ginger oil (GO) Banana peels oil (BPO) Time + 1.9529378345842E-05 Turmeric oil (TUO) Time² + 1.3750225211118E-05 Ginger oil (GO) Time² + 9.3431755704306E-06 Banana peels oil (BPO) Time² -0.084923738172294 Turmeric oil (TUO) Ginger oil (GO) Banana peels oil (BPO) Time -9.614175502662E-06 Turmeric oil (TUO) Ginger oil (GO) Time² -1.1150210766389E-05 Turmeric oil (TUO) Banana peels oil (BPO) Time² -5.4270270487429E-06 Ginger oil (GO) Banana peels oil (BPO) Time² + 0.0005771445757278 Turmeric oil (TUO) Ginger oil (GO) Banana peels oil (BPO) Time² | 0.0159734 | 3.1542857 | 0.5064029 | 0.9998414 | 0.999388 | 0.5817729 | 132.9577 |
| Iodine value | Iodine value = 65.392525201995 + 0.041324586535692 Time -0.0012561673092438 Time² | 1.4896005 | 57.99 | 2.5687195 | 0.9730466 | 0.97089 | 0.9658345 | 45.477627 |
| Carotenoids | Carotenoids = 747.50994567326 + 1.007170935196 Time -0.03566703639029 Time² + 0.00013015812960807 Time³ | 19.904032 | 660.9675 | 3.011348 | 0.9520383 | 0.946043 | 0.9325363 | 28.416533 |
| Tocopherols | Tocopherols = 205.64218840094 Turmeric oil (TUO) + 206.16844691325 Ginger oil (GO) + 205.90965783699 Banana peels oil (BPO) + 0.16801133300198 Turmeric oil (TUO) * Ginger oil (GO) + 0.60390516409581 Turmeric oil (TUO) * Banana peels oil (BPO) + 0.026829329062916 Turmeric oil (TUO) * Time -0.25345464831028 Ginger oil (GO) * Banana peels oil (BPO) -0.28153791945171 Ginger oil (GO) * Time -0.080838313998588 Banana peels oil (BPO) * Time -310.41044563233 Turmeric oil (TUO) * Ginger oil (GO) * Banana peels oil (BPO) -0.11600679520161 Turmeric oil (TUO) * Ginger oil (GO) * Time -0.51071772268267 Turmeric oil (TUO) * Banana peels oil (BPO) * Time -0.091037593954821 Ginger oil (GO) * Banana peels oil (BPO) * Time -0.0027819255436217 Turmeric oil (TUO) * Time² -0.00096603405856095 Ginger oil (GO) * Time² -0.002474695076716 Banana peels oil (BPO) * Time²+ 13.844336734749 Turmeric oil (TUO) * Ginger oil (GO) * Banana peels oil (BPO) * Time + 0.00058992918428513 Turmeric oil (TUO) * Ginger oil (GO) * Time² + 0.0031373374529964 Turmeric oil (TUO) * Banana peels oil (BPO) * Time² + 0.00042445860531682 Ginger oil (GO) * Banana peels oil (BPO) * Time² -0.093424613050445 Turmeric oil (TUO) * Ginger oil (GO) * Banana peels oil (BPO) * Time² | 2.1732797 | 711.8075 | 0.3053185 | 0.99988 | 0.999537 | 0.5173613 | 150.00413 |
| Specific viscosity | Specific viscosity = 32.797097551184 -0.11993175431554 Time | 2.0063006 | 23.930714 | 8.3837892 | 0.9166963 | 0.913492 | 0.898454 | 33.550072 |
| Oxidative stability index (OSI) | Oxidative stability index (OSI) = 47.015299846893 -0.059785896736247 Time -0.00082347314229505 Time² | 2.138688 | 35.74 | 5.984018 | 0.9608462 | 0.957714 | 0.9507946 | 39.277239 |
| Breakdown voltage | Free fatty acid content = 9.5442936095095 Turmeric oil (TUO) + 9.1729396708551 Ginger oil (GO) + 8.206389810888 Banana peels oil (BPO) -0.033213192149184 Turmeric oil (TUO) * Time -0.043311348710409 Ginger oil (GO) * Time -0.022648551661614 Banana peels oil (BPO) * Time -4.3778271858449e-06 Turmeric oil (TUO) * Time² + 4.7463319825557e-05 Ginger oil (GO) * Time² -6.9118832877871e-05 Banana peels oil (BPO) * Time² | 1.0874588 | 25.805 | 4.21414 | 0.9860863 | 0.980228 | 0.9746632 | 40.811302 |
| Smoke point | Smoke point = 304.72023525417 -0.56247946309232 Time + 0.0052178626094081 Time² -2.9384695214553e-05 Time³ | 4.9879074 | 275.88857 | 1.8079427 | 0.9612013 | 0.956351 | 0.9457828 | 35.084628 |
| A is Turmeric oil (TUO), B is Ginger oil (GO), C is Banana peels oil (BPO), D is Time | |  |  |  |  |  |  |  |
